# Multiclass Determination of 87 Mixed Veterinary Drugs, Pesticides and Mycotoxin Residues in Beef Muscle Samples by Ionic Liquid-Based Dispersive Liquid–Liquid Microextraction and Liquid Chromatography Tandem Mass Spectrometry

**DOI:** 10.3390/foods14050720

**Published:** 2025-02-20

**Authors:** Sandy O. S. Mookantsa, Simiso Dube, Mathew M. Nindi

**Affiliations:** 1 Department of Chemistry, College of Science, Engineering and Technology, The Science Campus, University of South Africa, Florida Park, Roodepoort, South Africa; 50810162@mylife.unisa.ac.za (S.O.S.M.); dubes@unisa.ac.za (S.D.); 2 Residue Section, Botswana National Veterinary Laboratory, Gaborone, Botswana; 3 Institute for Nanotechnology and Water Sustainability (iNanoWS), The Science Campus, College of Science, Engineering and Technology (CSET), University of South Africa, Florida Park, Roodepoort, South Africa

**Keywords:** ionic liquid-based dispersive liquid–liquid microextraction, sample preparation, liquid chromatography tandem mass spectrometry, multiclass, mixed contaminants, veterinary drugs, pesticides, mycotoxins, residues, green analytical chemistry metrics

## Abstract

A miniaturised, eco-friendly and efficient multiclass method for the simultaneous determination of 87 veterinary drugs, pesticides and mycotoxin residues in beef muscle samples by ionic liquid-based dispersive liquid–liquid microextraction (IL–DLLME) and liquid chromatography tandem mass spectrometry (LC–MS/MS) was developed and validated according to Commission Implementing Regulation (EU) 2021/808 and ISO/IEC 17025: 2017. Under IL–DLLME optimum conditions, matrix calibration yielded a coefficient of determination (*R*^2^) ranging from 0.99942 to 0.99997. The limit of detection (LOD) and limit of quantification (LOQ) ranged from 0.93 to 23.78 µg kg^−1^ and from 1.98 to 38.27 µg kg^−1^, respectively. Recoveries ranged from 80.0 to 109.8% and the decision limit (CCα) values ranged from 13.0 to 523.0 µg kg^−1^. Repeatability and reproducibility values were achieved in the ranges of 1.55–12.91% and 1.44–13.35%, respectively. The validated method was applied to 50 real beef samples and 12% of the tested samples contained traces of some residues, but they were all below their respective LOQs and CCα; hence, the beef was fit for human consumption. The greenness of the method was assessed using five green analytical chemistry (GAC) metrics, namely, the Analytical Eco-Scale (AES), NEMI, GAPI, AGREE and ComplexGAPI, and found to be green according to the AES metric and Analytical GREEnness Metric Approach and Software (AGREE). The method provided better results at a greatly reduced cost and analysis time in comparison with standard method.

## 1. Introduction

The administration of veterinary drugs and pesticides in food-producing animals and crops, respectively is crucial to prevent and treat diseases and promote growth [1,2]. Mycotoxins are naturally occurring secondary toxic fungal metabolites that may contaminate various agricultural products, including animal feed [3,4]. Inappropriate use or failure to observe withdrawal periods of veterinary drugs, as well as the ingestion of crops (feed) or water contaminated with mycotoxins and/or pesticides by food-producing animals, may result in the residues of these compounds or their metabolites being present in meat destined for human consumption [3,4,5]. The presence of these residues in meat may pose potential health risks to consumers such as resistance to antimicrobials and anthelmintics, allergic reactions, general toxic effects, immune system suppression, mutations, cancer and infertility [4,6,7]. To safeguard human health, the European Union (EU) and other food safety regulating agencies have established safe maximum residue levels (MRLs) and maximum permitted residue limits (MPRLs) for residues of veterinary drugs, hormones, pesticides and mycotoxins in animal tissues entering the human food chain [8,9,10].

The increasing vigilance and demand for the control of these residues in livestock and their primary products relies on effective residue monitoring plans. This is only made possible by having adequate technical resources and capabilities to implement the relevant legislation to control residues. The analytical methodology used must have an acceptable quality assurance supported by good quality control criteria, sufficient reference materials and technical data. Therefore, in the framework of analysing animal tissues destined for human consumption, it is imperative to develop test methods capable of identifying and quantifying all such substances explicitly. Several techniques are used to analyse these compounds, notably, ELISA, Charm II, HPLC–UV/FLD, GC–MS/MS and LC–MS/MS [1,3,4,11,12,13,14].

The ever-increasing demand for determining many compounds simultaneously at trace concentration levels in samples with complex matrices requires the use of analytical techniques such as LC–MS/MS and GC–MS/MS, which are characterised by a high sensitivity, selectivity, resolution and capacity to handle diverse classes of veterinary drugs and/or contaminant residues (>100 analytes) rapidly (<10.0 min) [1,2,4,13,14]. To take advantage of the multiresidue analysis capability of the LC–MS/MS technique, sample preparation methods should be incorporated to effectively extract a broad range of compounds from food matrices. The different physicochemical characteristics of the target analytes, as well as the presence of high concentrations of fat and proteins in the food matrices, complicate sample extraction and clean-up. Ideally, sample preparation methods of choice must also satisfy the requirements of green analytical chemistry (i.e., should be friendly to the environment by generating miniscule organic solvent waste or none) [15,16,17,18]. Most recently, a significant number of successful analytical sample extraction methods that use ionic liquids (ILs) in combination with dispersive liquid–liquid microextraction (DLLME) have been proposed. These methods combine the inherent advantages of ILs, such as low water solubility, high extraction efficiency for target compounds, low vapour pressure, having no effect on chromatographic behaviour and lower toxicity, with the intrinsic advantages of DLLME, such as the low consumption of reagents, high pre-concentration factors and speed [17,19,20,21,22].

Several publications have reported on multiresidue analysis in recent years. Although these methods can determine hundreds of veterinary drug and/or contaminant residues simultaneously, they generally focus on only one or two classes with similar analyte diversity (physicochemical properties and stability) and matrix diversity (matrix composition, analyte–matrix interactions and matrix co-extractives) [23,24,25]. To the best of our knowledge, there are a few studies dealing with the analysis of various classes of veterinary drugs and/or contaminants but they are focused on the optimisation of LC–MS/MS without assessing the efficiency and greenness of miniaturised sample treatment methods. The authors pointed out the difficulties of this approach, since the structural diversity of target compounds leads to heterogeneous behaviours during the LC–MS/MS analysis, both in terms of co-elution and signal suppression or enhancement due to retention and matrix effects, respectively [3,4,23,24,26]. Consequently, developing a multiclass method for the determination of mixed contaminant residues, capable of simultaneously analysing veterinary drugs and contaminants having a wide range of chemical structures and properties, remains a challenge, particularly in real food matrices due to their complexity.

This work focused on overcoming the above-mentioned challenges by developing multiclass analytical methods for residues which combine DLLME with ILs, as well as using stable isotope-labelled (or deuterium-labelled) internal standards (ISs) and matrix-matched standards. A multiclass method (13 classes) was developed and validated for the simultaneous determination of 87 veterinary drugs (quinolones, cephalosporins, macrolides, tetracyclines, sulphonamides, lincosamides, sedatives, Non-steroidal anti-inflammatory drugs (NSAIDs), anthelmintics, anticoccidials and avermectins), pesticides and mycotoxins (Table 1) in beef muscle samples, which uses the “green analytical chemistry” sample preparation/extraction (IL–DLLME) method combined with liquid chromatography tandem mass spectrometry (LC–MS/MS). Recently, there has been an increased awareness of the importance of the greenness of the analytical chemistry procedures and/or developed methods by analytical chemists. Several sample preparation strategies, such as miniaturisation in liquid-phase microextraction (LPME), have contributed to the movement towards greener analytical chemistry sample processes. Several green analytical chemistry (GAC) metrics have been developed to assess the greenness of the developed analytical chemistry methods. The greenness of this method (IL–DLLME/LC–MS/MS) was assessed by using five GAC metrics, namely, the Analytical Eco-Scale (AES), NEMI, GAPI, AGREE and ComplexGAPI.

## 2. Experimental Section

### 2.1. Reagents and Chemicals

Water (LiChrosolv^®^ LC–MS grade), acetonitrile (HPLC grade), methanol (HPLC grade), acetone (HPLC grade), dimethyl sulphoxide (DMSO, HPLC grade) and formic acid (ACS reagent, ≥98.0% HPLC grade) were sourced from Sigma-Aldrich (Steinheim, Germany). The three ionic liquid solvents, 1-butyl-3-methylimidazolium hexafluorophosphate ([C_4_MIm][PF_6_], ≥97.8% HPLC grade), 1-hexyl-3-methylimidazolium hexafluorophosphate ([C_6_MIm][PF_6_], ROTH, ≥99.0% HPLC grade) and 1-methyl-3-octylimidazolium hexafluorophosphate ([C_8_MIm][PF_6_], ≥95.0% HPLC grade), were donations received from the International Atomic Energy Agency (IAEA, Vienna, Austria). Sodium hydroxide anhydrous (NaOH, reagent grade, ≥98.0%) and sodium chloride (NaCl, ACS reagent, ≥99.5%) were sourced from Rochelle Chemicals and Lab Equipment cc (Johannesburg, South Africa). Water (deionised) was purified by a Milli-Q^®^ Direct Water Purification System from Merck Millipore (Darmstadt, Germany).

### 2.2. Laboratory Apparatus

The laboratory apparatuses used were the Heraeus Megafuge™ centrifuge, sourced from Thermo Scientific (Steingrund, Germany), the Ultra Turrax T25 homogeniser from Optolabor (Atlanta, GA, USA), the TurboVap LV Evaporator Concentration Workstation from Caliper Life Sciences (Hopkinton, MA, USA), the Heidolph™ Multi Reax Vortex Mixer (Heidolph Scientific Products GmbH, Schwabach, Germany) and 0.45 μm syringe filters with PVDF membrane from Pall Corporation (New York, NY, USA).

### 2.3. Analytical Instrument

**Figure 1:**
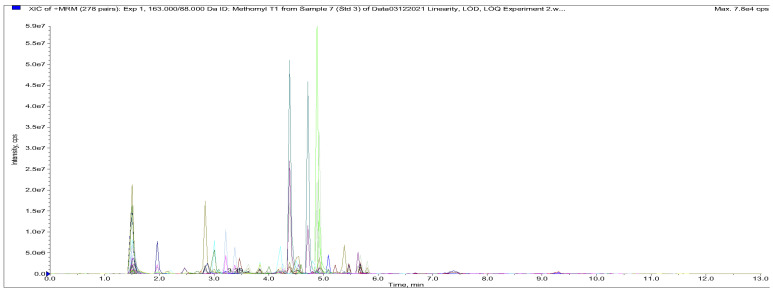
Chromatogram of a blank beef muscle sample fortified with 87 veterinary drug, pesticide and mycotoxin analytes at *MRLs*.

An LC–MS/MS instrument from AB Sciex LLC, Framingham, MA, U.S.A consisting of an AB SCIEX ExionLC^TM^ system and SCIEX QTRAP^®^ 6500^+^ mass spectrometer with the IonDrive^TM^ Turbo V source ESI was used in this work for the detection and quantification of the target analytes. The LC–MS/MS system was controlled by MasterView^TM^ Analyst^®^ software version 1.7 and the results were processed using MultiQuant^TM^ 3.0.2 software developed by AB SCIEX. The separation was achieved on the Phenomenex Kinetex 2.60 μm XB-C18 with 100 Å, a 100 × 4.60 mm LC column using mobile phase A: 0.1% formic acid in water (LC–MS grade) and mobile phase B: 0.1% formic acid in acetonitrile (LC–MS grade) at a flow rate of 0.550 mL min^−1^, an oven temperature of 40.0 °C and a 5.0 μL injection volume. The LC elution programme was as follows: (1) 0–1.00 min, 90.0% A; (2) 1.10–3.00 min, 90.0% A; (3) 3.10–8.00, 70.0% A; (4) 8.10–13.0, 90.0% A. The LC elution programme and optimised MS (ion source and gas) parameters are presented in Tables S1 and S2, respectively. A typical ion chromatogram is shown on Figure 1, with a good run time of <13.0 min.

### 2.4. Analytical Reference Standards

Analytical reference standards of veterinary drugs (tetracyclines, sulphonamides, lincosamides, macrolides, quinolones, sedatives, anthelmintics, cephalosporins, NSAIDs, ivermectin and anticoccidials) and pesticides were all of ≥98.0% (HPLC) purity and obtained from Sigma-Aldrich (Steinheim, Germany). Mycotoxin standards (ochratoxin A and zearalenone, with a 97.0% and 99.5% (HPLC) purity, respectively) were supplied by R-Biopharm AG (Darmstadt, Germany). The deuterium/isotope-labelled standards (tetracycline-d6 (TTC-d6), sulphamethazine-d4 (SFT-d4), sulphapyridine-d4 (SFP-d4), lincomycin-d3 (LCM-d3), spiramycin-d3 (SRM-d3), enrofloxacin-d5 (EFX-d5), difloxacin-d5 (DFC-d5), xylazine-d6 (XLZ-d6), mebendazole-d3 (MBZ-d3), albendazole-d3 (ABZ-d3), triclabendazole-d3 (TCZ-d3), ceftiofur-d3 (CTF-d3), flunixin-d3 (FLX-d3), carbaryl-d7 (CBL-d7), ochratoxin A 13C20 (OCT-13C20) and zearalenone (ZRL-13C18)) were all ≥97.0% (HPLC) pure and sourced from Toronto Research Chemicals Inc. (Toronto, ON, Canada).

### 2.5. Preparation of Analytical Standard Solutions

Analytical stock standard solutions of every compound were prepared separately in methanol, methanol/DMSO and acetonitrile solvents depending on the solubility of the compound/analyte at a concentration of 1.00 mg mL^−1^. Most of these stock solutions were prepared by weighing an amount corresponding to 10.0 ± 0.200 mg (depending on the substance purity, water and salt content of the compound), quantitatively transferring it to a 10.0 mL volumetric flask and making up to the mark with the relevant solvent. The working standard solutions were prepared such that, when 2.00 g of the beef muscle sample was spiked with 200.0 μL of standard aliquot, the concentration in the sample was equivalent to the MRL of the substance/analyte. The volumetric flask was filled up to the mark with methanol and stored at −30.0 ± 10.0 °C.

### 2.6. Procedures

#### 2.6.1. Sampling

Sampling was performed according to European Commission Regulation (EU) 2017/625 of 15 March 2017 (also known as the Official Controls Regulation (OCR)), and Annex II of Commission Implementing Regulation (EU) 2021/808 of 22 March 2021, laying down the sampling procedures and the official sample treatment for residues of pharmacologically active substances in food-producing animals [27,28]. These regulations state that samples shall be obtained, handled and processed according to national residue control programme in such a way that sample integrity and traceability are maintained, and the minimum quantities shall be sufficient to enable the laboratory to carry out the analytical procedures necessary to complete the screening and confirmatory analyses. The Botswana National Veterinary Laboratory compiled a detailed sampling guide which was used at Botswana Meat Commission abattoirs (Lobatse, Francistown and Maun) and municipal abattoirs around the country to sample bovine (beef) muscles (50.0 g × 100) for this study. Residues of veterinary drugs, pesticides and mycotoxins were distributed to the liver, muscle, kidney and fat following absorption from the gastro-intestinal system or injection site after administration of these drugs to animals or ingestion of contaminants; hence, the choice of muscle/meat samples (most commonly consumed bovine (beef) product) for this study.

#### 2.6.2. Sample Preparation

##### Isolation of Residues from Beef Muscle Samples

Isolation of residues from beef muscle samples was carried out using a sample pre-treatment procedure previously described by Mookantsa and co-workers [21], with minor improvements. Briefly, 2.00 ± 0.0500 g of previously homogenised beef muscle samples were weighed into 50.0 mL polypropylene centrifuge tubes. For quality control, three (3) extra pre-extraction matrix spike (PrEMS) samples were fortified with working standard solution at 1.00 × MRL concentration. Six (6) additional pre-extraction matrix spike (PrEMS) samples were fortified with standard solution at 0.1, 0.25, 0.5, 1.0, 1.5 and 2.0 × MRL concentrations for the matrix-matched calibration. A 50.0 μL solution of mixed stable isotope-labelled (or deuterium-labelled) internal standards (ISs) was added to all of the samples, controls and calibrations. After fortification, the samples were allowed to stand for a minimum time of 5.00 min before extraction. A solution of 6.00 mL of acetonitrile and 2.00 mL of water was added to all of the samples using a bottle-top dispenser (BTD) and mixed vigorously for 5.00 min on a vortex mixer, followed by centrifugation at 2795× *g* for 5.00 min. The supernatant was collected and concentrated to nearly 100.0 μL using a stream of nitrogen gas in the Caliper TurboVap LV Evaporator Concentration Workstation, which was set at 50.0 °C. The concentrated solution was diluted with 5.00 mL of water, filtered through 0.45 μm syringe filters into 15.0 mL conical-bottom centrifuge tubes, and the pH was adjusted to 6.00 using NaOH 0.100 M and 0.1% formic acid prior to the IL–DLLME procedure.

##### IL–DLLME Procedure

The disperser solvent (0.400 mL of acetonitrile) containing 60.0 μL of ionic liquid (1-octyl-3-methylimidazolium hexafluorophosphate ([C_8_MIm][PF_6_])) was quickly injected into the sample extract. The ternary component solvent system was mixed immediately by a vortex mixer for 30.0 s, allowed to stand for 60.0 s and then centrifuged at 2795× *g* for 5.00 min. The upper aqueous phase was discarded, and the sedimented ionic liquid phase, laden with analyte residues, was diluted with 200.0 μL of acetonitrile/water, 5.0 μL of which was injected into the LC–MS/MS system.

##### Procedure for Optimisation of IL–DLLME Parameters

The IL–DLLME parameters were optimised by first selecting the ionic liquid and the disperser solvent. Among the ionic liquid (IL) extraction solvents, 1-octyl-3-methylimidazolium hexafluorophosphate [C_8_MIm][PF_6_] (density, 1.23 g mL^−1^), 1-hexyl-3-methylimidazolium hexafluorophosphate [C_6_MIm][PF_6_] (density, 1.30 g mL^−1^) and 1-butyl-3-methylimidazolium hexafluorophosphate [C_4_MIm][PF_6_] (density, 1.38 g mL^−1^) were studied [25]. In this work, 50.0 µL of each of the IL extraction solvents and 500.0 µL of acetonitrile were added to the sample extracts. The IL–DLLME procedure was followed and the sedimented phases were dissolved in acetonitrile/water (2:1), and 5.0 µL was injected into the LC–MS/MS system.

Methanol, acetone and acetonitrile were investigated as disperser solvents, and the effect of these solvents on the performance of the DLLME procedure was assessed. The experiments were performed using the sample extract, 50.0 µL of ionic liquid ([C_8_MIm][PF_6_]) and 0.500 mL of either methanol, acetone or acetonitrile. The IL–DLLME procedure was followed, the analytes in the sedimented phase were dissolved in acetonitrile/water and then injected into the LC–MS/MS system. The influence of the pH of the sample extract on the recovery of mixed contaminants (veterinary drug, pesticides and mycotoxin) analytes was investigated at a pH range of 3.00–9.00 by using a formic acid and sodium hydroxide solution to adjust the pH while keeping all of the other parameters constant (i.e., acetonitrile (0.500 mL) and ionic liquid ([C_8_MIm][PF_6_]) (50.0 µL) as disperser and extraction solvents, respectively).

The effect of the volume of the disperser solvent was investigated by using different volumes of acetonitrile (0.300, 0.400, 0.500, 0.600, 0.800 and 1.00 mL) at a constant volume of 50.0 µL of ionic liquid (1-octyl-3-methylimidazolium hexafluorophosphate) and maintaining the sample extract at a pH of 6.00. The effect of the volume of the IL extraction solvent was investigated by keeping the acetonitrile volume at the optimum of 0.400 mL and sample extracts at a pH of 6.00, while varying the volumes of the 1-octyl-3-methylimidazolium hexafluorophosphate (40.0, 50.0, 60.0, 70.0, 80.0 and 90.0 µL). The IL–DLLME procedure was followed for all of the sample extracts and they were analysed as described above. Other parameters that were investigated in the same manner included the centrifugation time, extraction time and salt (NaCl) addition.

#### 2.6.3. IL–DLLME Method Validation

The Commission Implementing Regulation (EU) 2021/808 of 22 March 2021 (European Commission, 2021) [28], which defines the performance criteria for the validation of analytical methods, and International Standard ISO/IEC 17025: 2017 [29], which specifies the general requirements for the competence of testing and calibration laboratories, were used for the validation of the IL–DLLME method. Several validation parameters, such as the linearity, recovery, repeatability, reproducibility, limit of detection (LOD), limit of quantification (LOQ) and decision limit (CCα), were determined under optimal conditions. Stable isotope-labelled standards or deuterium-labelled standards corresponding to individual analytes were used as internal standards (ISs). An equal volume (50.0 µL) of the IS was added to all of the samples, and the ratio of peak area of the analytes to the peak area of the internal standard was used in the quantification of the analytes, namely, veterinary drugs, pesticides and mycotoxins, in all of the IL–DLLME method validation experiments.

##### Procedure for Linearity

Linearity was determined by fortifying blank beef muscle samples with the analyte standard mixture at concentrations corresponding to six calibration levels in the range of 1.50–1000 µg kg^−1^ as per the analyte MRL at 0.1, 0.25, 0.5, 1.0, 1.5 and 2.0 × MRL. Triplicate samples containing the analyte at the six different calibration levels were analysed using the entire IL–DLLME procedure and used to construct the calibration curves (n = 3). The obtained data were used to calculate the coefficient of determination (*R*^2^), the limit of detection (LOD) and the limit of quantification (LOQ).

##### Procedure for IL–DLLME Validation

The validation study was carried out using blank beef muscle samples that were spiked at three different concentration levels (0.5, 1.0 and 1.5 × MRL) of individual analytes, and the analysis was performed at each level with seven replicates on three separate occasions. The samples that were used to prepare the validation batch were screened prior to the procedure and found negative for the target analytes (blank samples). The experiment was repeated on two occasions (two consecutive weeks) to make a total number of 21 samples per spiking level (n = 21). The data obtained from the three (3) validation batches were used to calculate validation parameters such as the decision limit (CCα), reproducibility and repeatability for the IL–DLLME method.

##### Procedure for Trueness/Accuracy Studies

A blank beef muscle sample was spiked with mixed veterinary drug, pesticide and mycotoxin analyte standards at a range of concentrations (0.1, 0.25, 0.5, 1.0, 1.5 and 2.0 × MRL) and used to construct a matrix-matched calibration curve for each compound. Also, blank beef muscle samples were spiked at three concentration levels (0.5, 1.0 and 1.5 × MRL) of each analyte and the analysis was performed at each level with seven replicates on three separate occasions. All of these spiked samples were extracted using the IL–DLLME method. The data obtained from these experiments were used to estimate the overall method percentage recovery, relative standard deviation and reproducibility at each spiking level.

##### Application of IL–DLLME on Real Beef Meat Samples

The validated IL–DLLME method was applied to 50 real beef meat samples for the determination of veterinary drug, pesticide and mycotoxin residues to determine their presence and prevalence below or above the MRLs. The beef meat samples were obtained from national abattoirs, local butcheries and supermarkets in Botswana.

## 3. Results and Discussion

### 3.1. Optimisation of the IL–DLLME Extraction

The study reports on the IL–DLLME multiclass (13 classes) method for the extraction of veterinary drug, pesticide and mycotoxin residues in beef muscle samples. In this study, the most important factors affecting the extraction recoveries of target analytes in IL–DLLME methods, namely, the ionic liquid solvent and its volume, disperser solvent and its volume, extraction and centrifugation time, pH and salt addition, were investigated by using the one-factor-at-a-time (OFAT) approach. For simplicity in this work, two (2) representative analytes per class and extraction recoveries (ERs) were used to optimise the above-mentioned parameters. Extraction recovery (ER) is defined as the percentage of the total analyte amount (*n*_0_) extracted to the sedimented phase (*n*_sed_), calculated as ER = *n*_sed_/*n*_0_ × 100 [30]. The experiments were carried out according to the sample preparation procedure described above in Section 2.6.2.

#### 3.1.1. Selection of Ionic Liquid

**Figure 2:**
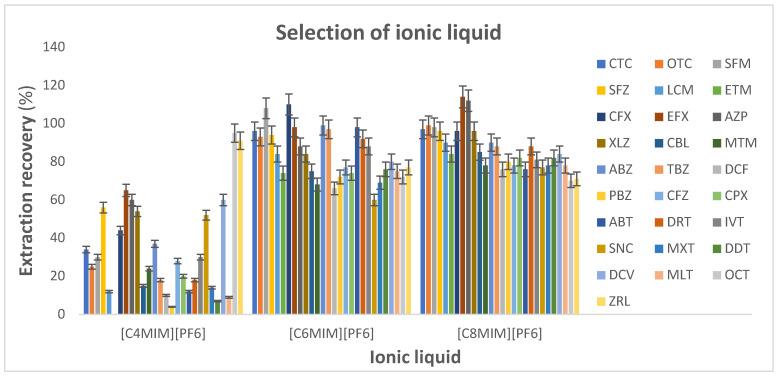
Selection of the ionic liquid (blank extracts: 5.0 mL; spiking level: 1 MRL; disperser solvent (acetonitrile): 0.5 mL; extraction time: 1.0 min; centrifugation time: 5.0 min; ionic liquid ([C_4_MIm][PF_6_], [C_6_MIm][PF_6_] and [C_8_MIm][PF_6_]): 50 µL).

In an IL–DLLME method, the appropriate IL extraction solvents should meet minimum requirements, such as a low solubility in water, a higher density than water, good extraction ability for the target analytes, miscible with the disperser solvent and have no effect on the chromatographic behaviour. Based on these considerations, three ILs, namely, [C_4_MIm][PF_6_], [C_6_MIm][PF_6_] and [C_8_MIm][PF_6_], were investigated. Figure 2 shows that an increase in extraction recoveries was observed with the increase in the alkyl chain length of imidazole ions, such that highest extraction recoveries (70.0–120%) were achieved with [C_8_MIm][PF_6_], which had the lowest solubility in water relative to the investigated IL extraction solvents. The solubility of [C_4_MIm][PF_6_], [C_6_MIm][PF_6_] and [C_8_MIm][PF_6_] in water is 18.8, 7.5 and 2.0 mg L^−1^, respectively [18]. Hence, [C_8_MIm][PF_6_] was used as an ionic liquid extraction solvent for all of the subsequent experiments in this study.

#### 3.1.2. Selection of Disperser Solvent

**Figure 3:**
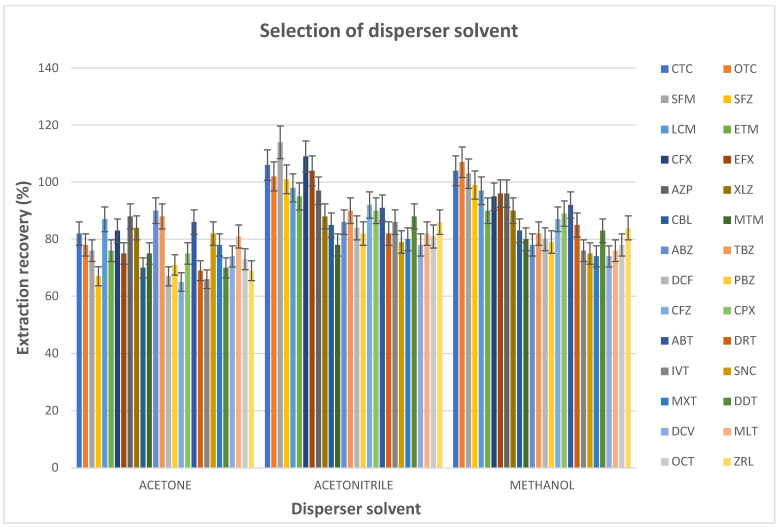
Selection of the disperser solvent (blank extracts: 5.0 mL; spiking level: 1 MRL; ionic liquid ([C_8_MIm][PF_6_]): 50 µL; extraction time: 1.0 min; centrifugation time: 5.0 min; disperser solvent (acetonitrile, methanol and acetone): 0.5 mL).

The selection of the disperser solvent is also crucial for the IL–DLLME method. The disperser solvent should be miscible with both the aqueous phase and IL extraction solvent for the formation of a cloudy solution, and should effectively dissolve the target analytes [18,31]. In this study, methanol, acetonitrile and acetone were evaluated as disperser solvents. All three solvents performed well as disperser solvents, with extraction recoveries of >60.0% (Figure 3). However, acetonitrile yielded the highest recoveries (>75.0%) for all of the target analytes; hence, it was selected for all of the subsequent studies.

#### 3.1.3. Optimisation of the pH of Aqueous Phase

**Figure 4:**
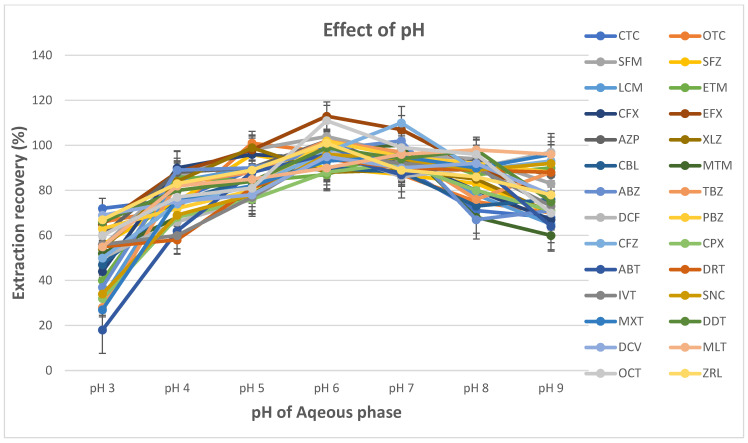
Effect of pH of aqueous blank extracts on the extraction recovery (blank extracts: 5.0 mL; spiking level: 1 MRL; disperser solvent (acetonitrile): 0.4 mL; ionic liquid ([C_8_MIm][PF_6_]: 60 µL; extraction time: 1.0 min; centrifugation time: 5.0 min; pH: 3, 4, 5, 6, 7, 8 and 9).

Most of the target compounds in this study are amphoteric; hence, they are charged over a wide pH range. The pH is a critical parameter because it determines the existing state of analytes and their extraction recovery capabilities. An appropriate pH would allow most of the analytes to be extracted from the aqueous phase to the IL extraction solvent in their neutral form. In this work, the effect of pH was studied in the range of 3.00–9.00. Figure 4 shows that the extraction recoveries increased gradually in the pH range of 3.00–6.00 and decreased from a pH of 7.00–9.00. The highest recoveries (>80.0%) were observed at a pH of 6.00; hence, a pH of 6 was selected as the optimum pH for the subsequent experiments.

#### 3.1.4. Optimisation of Ionic Liquid ([C_8_MIm][PF_6_]) Extraction Volume

The effect of the volume of IL on the extraction efficiency was evaluated by varying the volume of [C_8_MIm][PF_6_] over the range of 40.0–90.0 µL, while all of the other experimental parameters were kept constant. The results shown in Figure S1 indicate that the ERs of the target analytes increased when the IL volume increased from 40.0 to 60.0 µL, and then decreased rapidly beyond this volume. This is because increasing the volume of IL allows for more of the extraction solvent to be sedimented, which increases the enrichment. However, once the volume of IL reaches a certain level, the enrichment effect is reduced because the analyte concentration in the sediment drops as the volume of extraction solvent increases [22]. Therefore, 60.0 µL was selected as the optimum volume of [C_8_MIm][PF_6_] for all of the subsequent experiments.

#### 3.1.5. Optimisation of Disperser Solvent Volume

The effect of the disperser solvent volume was examined by varying volumes of acetonitrile (0.3, 0.4, 0.5, 0.6, 0.8 and 1.0 mL) while maintaining the optimum volume (60 µL) of the ionic liquid [C_8_MIm][PF_6_] to determine the ERs of the target analytes. The experimental results in Figure S2 reveal that the ERs of all target analytes were higher (>85.0%) at an acetonitrile volume of 0.400 mL. A further increase in the acetonitrile volume from 0.400 mL to 1.00 mL resulted in lower ERs. The reason for this is that acetonitrile cannot disperse [C_8_MIm][PF_6_] effectively at a low volume and, therefore, the cloudy solution is not completely formed. On the other hand, at high volumes of acetonitrile, the solubility of the target analytes in water increases, leading to a decrease in ERs. In addition, when a large volume of disperser solvent is used, the IL can be dissolved in the disperser solvent, resulting in a low volume of sedimented IL phase or no formation of the sedimented phase after centrifugation. Based on the experimental results, 0.400 mL was selected as the optimum volume of acetonitrile.

#### 3.1.6. Effect of Salt (NaCl) Addition

Adding salt (e.g., NaCl) to a sample solution causes a salting-out effect, thus decreasing the solubility of target analytes into the aqueous sample solution and consequently promoting the transfer of analytes into the organic or IL phase. However, the high ionic strength in an IL–DLLME system can enhance the solubility of an IL in the aqueous phase, thereby decreasing the extraction performance [22]. In this work, various amounts of NaCl (0, 0.5, 1.0, 2.0, 4.0 and 6.0%, *w*/*v*) were added to the fortified sample extracts prior to the IL–DLLME procedure. High extraction recoveries (>80.0%) were obtained for all analytes when the concentrations of NaCl were 0, 0.5 and 1.0%, but decreased rapidly when the salt concentration exceeded 1.0% (Figure S3). Overall, no significant benefit of adding the salt was observed; hence, NaCl was not added in the subsequent experiments.

#### 3.1.7. Effect of Extraction Time

The extraction time is defined as the interval time between the addition of the IL and the centrifugation. Generally, the maximum quantity of analyte was transferred into the IL phase when the extraction equilibrium was reached [22,31]. In the investigation for the effect of the extraction time, different times (from 3.00 to 60.0 s) were explored by vortex mixing the ternary component solvent mixture to evaluate the optimum extraction time. The extraction efficiency in this study revealed that the extraction equilibrium was attained at 30.0 s, and a longer extraction time did not significantly affect the extraction efficiency (Figure S4). These results clearly showed that IL–DLLME is a very fast extraction process, with the extraction equilibrium being reached just after the cloudy solution was formed. The large surface area between the IL droplets and the aqueous phase, which is a key factor in facilitating the equilibrium, resulted in rapid extraction [22]. To ensure the maximum extraction efficiency for all analytes, the cloudy solution was left to stand for 60.0 s prior to centrifugation.

#### 3.1.8. Effect of Centrifugation Time

**Figure 5:**
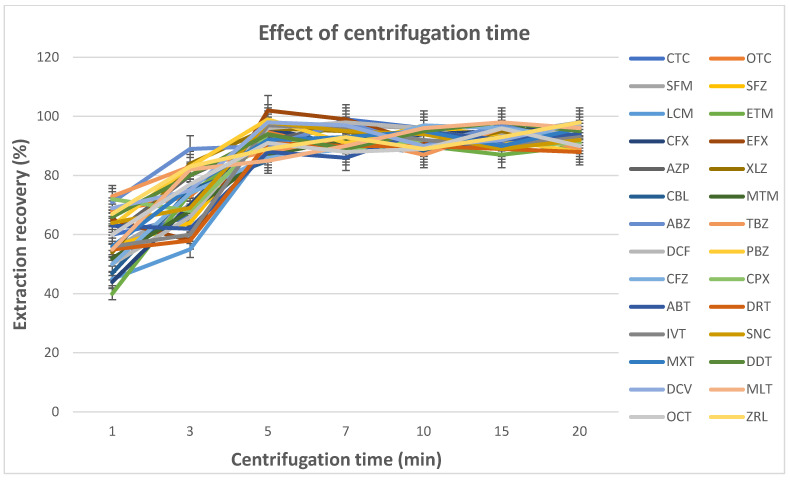
Effect of the centrifugation time on the extraction recovery (blank extracts: 5.0 mL; pH of 6; spiking level: 1 MRL; disperser solvent (acetonitrile): 0.4 mL; ionic liquid ([C_8_MIm][PF_6_]): 60 µL; extraction time: 1.0 min; centrifugation time: 1.0, 3.0, 5.0, 7.0, 10.0, 15.0 and 20.0 min).

The cloudy solution formed was centrifuged for the fine IL droplets to settle at the bottom of the centrifuge tube; however, it is important to establish the optimum centrifugation time with respect to the settled IL volume and the analyte concentration in the IL phase [18,22]. To evaluate the optimum time required for a complete phase separation, the centrifugation time was investigated in the range of 1.00–20.00 min at 2795× *g*. The results displayed in Figure 5 confirm that 5.00 min was sufficient for a complete separation. Therefore, 5.00 min was selected as the optimum centrifugation time.

### 3.2. IL–DLLME Method Validation

Under optimised conditions (0.4 mL of acetonitrile (dispersive solvent), 60 µL of [C_8_MIm][PF_6_] (extraction solvent), an extraction time of 30 s for vortex mixing and 60 s standing and a centrifugation time of 5.0 min), and at a pH of 6, the performance of the proposed method was evaluated by determining crucial validation parameters such as the linearity, LOD, LOQ, CCα, precision and accuracy by following the validation procedure described above in the Experimental Section 2.6.3.

#### 3.2.1. Validation Parameters (Linearity, LOD, LOQ and CCα)

The linearity of the method was obtained using the matrix-matched calibration curves for concentrations of 0.1, 0.25, 0.5, 1.0, 1.5 and 2.0 times MRL, with the coefficient of determination (*R*^2^) ranging from 0.99942 to 0.99997. The LODs and LOQs ranged from 0.93 to 23.78 µg kg^−1^ and 1.98 to 38.27 µg kg^−1^, respectively. The CCα values ranged from 13.0 to 523 µg kg^−1^ and were dependent on the MRL concentration levels. The calculated *R*^2^, LOD, LOQ and CCα values, based on inter-batch reproducibility data, are shown in Table 2.

#### 3.2.2. Precision (Repeatability and Reproducibility) and Accuracy

The repeatability of the method was determined by calculating the intra-day (within-batch) variability based on the relative standard deviation (%RSD) of seven (7) replicates of beef muscle samples spiked at 0.5, 1.0 and 1.5 × MRL levels of individual analytes injected by the same analyst within a 24-h period. Similarly, reproducibility (inter-day variability) was established as the %RSD of the samples prepared by two different analysts and injected over a period of two weeks. Repeatability and reproducibility values were obtained in the range of 1.55–12.91% and 1.44–13.35%, respectively. The inter-day variabilities were understandably a little higher than the intra-day variabilities. Accuracy values, calculated as the percentage recoveries of spiked blank beef muscle samples at three spiking levels (0.5, 1.0 and 1.5 × MRL), ranged from 80.0 to 109.8%. As shown in Table 3, the results complied with the set standards in Commission Implementing Regulation (EU) 2021/808 of March 2021 [28]. According to Commission Implementing Regulation (EU) 2021/808 of March 2021 (European Commission, 2021), the relative matrix effect should be determined either as part of the validation or in separate experiments. In this study, the evaluation of matrix effects was not performed during the validation but was significantly minimised by using matrix-matched standards and internal standards to calibrate the analysis.

#### 3.2.3. Comparison of IL–DLLME with QuEChERS Methods

The validated IL–DLLME method was compared with standard QuEChERS methods by using a paired t-test at the 95% confidence level. The QuEChERS methods were validated by the Botswana National Veterinary Laboratory and accredited by the Southern African Development Community Accreditation Service (SADCAS). Mean recoveries of the studied analytes for the two analytical methods were compared at concentration levels of 0.5, 1.0 and 1.5 × MRL. Ochratoxin A (OCT) and zearalenone (ZRL) were not validated in beef meat samples using the QuEChERS method, and the results obtained for these two target analytes using the IL–DLLME method could thus not be compared. The results in Table S3 show that the calculated t-value for all analytes is less than the t-critical value of 4.30, and hence the results for the two methods do not differ significantly in terms of the accuracy and reproducibility. The two methods were also compared in terms of time, resources and costs (Table S4). The IL–DLLME multiclass method was found to be cheaper, more efficient and ecologically friendly in terms of the costs of the reagents used, the sample preparation and analysis time and the quantity of the reagent waste [25,32].

#### 3.2.4. Application of the IL–DLLME Method on Real Beef Meat Samples

Finally, at the Botswana National Veterinary Laboratory, the validated method was applied successfully to 50 real beef meat samples sourced from Botswana abattoirs, butcheries and supermarkets for the analysis of veterinary drug, pesticide and mycotoxin residues. The results showed that 12.0% of the tested samples contained traces of some veterinary drug residues, but they were all below their respective limit of quantification (LOQ) and decision limit for confirmation (CCα); hence, the meat was approved for human consumption.

### 3.3. Greenness Assessment of Developed IL–DLLME/LC–MS/MS Method

In this work, the greenness of the developed IL–DLLME/LC–MS/MS method was assessed using five GAC metrics, namely, the Analytical Eco-Scale (AES), National Environmental Methods Index (NEMI), Green Analytical Procedure Index (GAPI), Analytical GREEnness (AGREE) Metric Approach and Software and ComplexGAPI. The greenness profile of the developed IL–DLLME/LC–MS/MS method, evaluated using these five GAC metrics, is indicated in Table 4.

The National Environmental Methods Index (NEMI) was one of the earliest tools for assessing the greenness of analytical chemistry methods [33]. Although the NEMI metric was first developed for water quality and environmental monitoring, it now extends to other matrices such as soil or sediment and biological matter. The greenness profile is indicated in the form of a pictogram with four quadrants relating to different assessment criteria; the quadrant is shaded green if the criteria are met and remains blank if the criteria are not met. Table 4 shows the NEMI pictogram of the developed IL–DLLME/LC–MS/MS method, indicating that three of the four quadrants are coloured green.

The Analytical Eco-Scale (AES) approach was developed by Gałuszka and co-workers as a tool for evaluating the greenness of analytical chemistry methods [34]. The tool is based on assigning penalty points to parameters of the analytical process that deviate from ideal green analysis, and the total penalty points are then subtracted from a maximum score of 100. An ideal green method would score 100, which is not usually possible; hence, a score >75.0 indicates excellent greenness, ≥50.0 to ≤75.0 indicates acceptable greenness and a score of <50.0 indicates inadequate greenness. Our developed method received a score of 85.0 on the Analytical Eco-Scale (AES), indicating that it is an excellent green method. More inclusive tools, namely, the Green Analytical Procedure Index (GAPI) [35] and Analytical GREEnness Metric Approach and Software (AGREE) [36], were then created by merging the benefits of quantitative scoring and visual illustration [36]. Table S5 gives a description and colour coding of the GAPI parameters, Table S6 gives the parameters and colour coding for the ComplexGAPI and Table 4 lists the scoring value of the AGREE assessment tool for the IL–DLLME/LC–MS/MS method. The GAPI tool is based on a pictogram consisting of colour-coded pentagrams, allowing for a quick review of the entire analytical chemistry methodology and providing a comprehensive overview of the greenness of a variety of fields. Table 4 shows that three (3) of the five (5) pentagrams were flagged red, which included sample collection, extraction and no treatment of waste (see Table S5). The developed method received an overall AGREE score of 0.670, and is therefore considered green (Table 4). According to the literature, an AGREE score of at least 0.600 needs to be attained for the method to be considered green [37]. Detailed explanations of these greenness tools can be found in Sajid [38].

Płotka-Wasylka and Wojnowski developed a complementary green analytical procedure index, ComplexGAPI, which includes software [39]. ComplexGAPI software was used to generate six (6) pictograms that allowed for the simple visual representation of the comprehensive analytical chemistry methodology. Similarly to the GAPI, pictograms use hexagons with fields corresponding to each step of the analytical chemistry methodology, and fields are coded green, yellow and red, depending on the low, medium and high environmental impact associated with each of the steps. However, in ComplexGAPI, an additional hexagonal field (*E*-factor) is added to the bottom of the GAPI pictogram, representing processes performed prior to the analytical chemistry method itself. Two of the six pictograms were flagged red, which were associated with the sample preparation and methods of analysis. As can be seen in Table S6, two (2) of the sample preparation fields were coloured red due to the off-line sample collection process and the extraction process required; in addition, the method category was also coloured red due to the types of analysis performed (i.e., qualitative and quantitative).

## 4. Conclusions

A cost-effective, efficient and environmentally friendly multiclass ionic liquid-based dispersive liquid–liquid microextraction method combined with LC–MS/MS was developed, validated and applied for the simultaneous determination of 87 mixed veterinary drug, pesticide and mycotoxin residues in spiked beef muscle samples. The effects of various parameters on the IL–DLLME method were evaluated and optimum conditions were established. Under the optimum conditions, the method was validated according to Commission Implementing Regulation (EU) 2021/808 of March 2021, and all of the parameters complied with the set standards. Compared with other standard methods, this multiclass method has several advantages such as a shorter analysis time, consumption of small volume of solvents (greener), wide linear range and the capability of simultaneously analysing larger numbers of analytes with a wide range of polarities. This work has shown that, with slight modifications, this multiclass method can probably be applied to other analytes, animal species and biological matrices. The greenness of the IL–DLLME/LC–MS/MS method was evaluated using five GAC metrics, and was found to be green according to the Analytical Eco-Scale (AES) metric and Analytical GREEnness Metric Approach and Software (AGREE) tools. Areas of improvement with respect to the greenness were highlighted by the GAPI, AGREE and ComplexGAPI tools.

## Figures and Tables

**Table 1:** Classification and identification of the studied compounds.

Compound	Abbreviation	Class	CAS Number	Molecular Weight (g mol^−1^)
Chlortetracycline	CTC	Tetracycline	64-72-2	478.11
Doxycycline	DXC	Tetracycline	24390-14-5	444.53
Oxytetracycline	OTC	Tetracycline	2058-46-0	460.15
Tetracycline	TTC	Tetracycline	64-75-5	444.15
Sulphamerazine	SFM	Sulphonamide	127-79-7	264.30
Sulfisoxazole	SFX	Sulphonamide	127-69-5	267.30
Sulphamethazine	SFT	Sulphonamide	57-68-1	278.33
Sulphadimethoxine	SFD	Sulphonamide	122-11-2	310.33
Sulphathiazole	SFZ	Sulphonamide	72-14-0	255.32
Sulphachloropyridazine	SFC	Sulphonamide	80-32-0	284.72
Sulphaquinoxaline	SFQ	Sulphonamide	967-80-6	322.32
Sulphapyridine	SFP	Sulphonamide	144-83-2	249.29
Sulphamonomethoxine	SFN	Sulphonamide	1220-83-3	280.30
Sulphamethizole	SFL	Sulphonamide	144-82-1	270.33
Sulphamethoxypyridazine	SFO	Sulphonamide	80-35-3	280.30
Sulphadiazine	SFA	Sulphonamide	68-35-9	250.28
Lincomycin	LCM	Lincosamide	859-18-7	406.54
Erythromycin	ETM	Macrolide	114-07-8	733.93
Tilmicosin	TMC	Macrolide	108050-54	869.13
Tylosin	TLS	Macrolide	8026-48-0	916.10
Spiramycin	SRM	Macrolide	8025-81-8	843.05
Gamithromycin	GTM	Macrolide	145435-72-9	777.04
Tulathromycin	TTM	Macrolide	217500-96-4	806.10
Ciprofloxacin	CFX	Quinolone	85721-33-1	331.34
Danofloxacin	DFX	Quinolone	112398-08-0	357.38
Difloxacin	DFC	Quinolone	98106-17-3	399.39
Enrofloxacin	EFX	Quinolone	93106-60-6	359.39
Flumequine	FMQ	Quinolone	42835-25-6	261.25
Norfloxacin	NFX	Quinolone	70458-96-7	319.33
Oxolinic acid	OXA	Quinolone	14698-29-4	261.23
Carazolol	CZL	Sedative	57775-29-8	298.38
Chlorpromazine	CPZ	Sedative	50-53-3	318.86
Acepromazine	APZ	Sedative	61-00-7	326.46
Xylazine	XLZ	Sedative	7361-61-7	220.34
Propionylpromazine	PPZ	Sedative	3568-24-9	340.49
Azaperol	AZP	Sedative	2804-05-9	329.41
Triclabendazole-sulphoxide	TCZ-SO	Anthelmintic	100648-13-3	375.66
Amino-mebendazole	MBZ-NH_2_	Anthelmintic	52329-60-9	237.26
Oxyclozanide	OXC	Anthelmintic	2277-92-1	401.46
Fenbendazole	FBZ	Anthelmintic	43210-67-9	299.35
Albendazole	ABZ	Anthelmintic	54965-21-8	265.33
Levamisole	LVS	Anthelmintic	14769-73-4	204.29
Clorsulon	CSL	Anthelmintic	60200-06-8	380.66
Oxibendazole	OBZ	Anthelmintic	20559-55-1	249.27
Triclabendazole	TCZ	Anthelmintic	68786-66-3	359.66
Thiabendazole	TBZ	Anthelmintic	148-79-8	201.25
Nitroxynil	NTX	Anthelmintic	1689-89-0	290.01
Closantel	CST	Anthelmintic	57808-65-8	663.07
Rafoxanide	RFX	Anthelmintic	22662-39-1	626.01
Albendazole-2-aminosulphone	ABZ-SO_2_-NH_2_	Anthelmintic	80983-34-2	239.29
5-Hydroxythiabendazole	TBZ-OH	Anthelmintic	948-71-0	217.25
Cephalexin	CPX	Cephalosporin	15686-71-2	347.39
Cefquinome	CFQ	Cephalosporin	84957-30-2	528.60
Cefazolin	CFZ	Cephalosporin	25953-19-9	454.51
Cephalonium	CFN	Cephalosporin	5575-21-3	458.51
Flunixin	FLX	NSAIDs	38677-85-9	296.25
Meloxicam	MXC	NSAIDs	71125-38-7	351.40
Diclofenac	DCF	NSAIDs	15307-86-5	296.15
Phenylbutazone	PBZ	NSAIDs	50-33-9	308.37
Tolfenamic acid	TFA	NSAIDs	13710-19-5	261.71
Ketoprofen	KTF	NSAIDs	22071-15-4	254.28
Ibuprofen	IBF	NSAIDs	15687-27-1	206.28
Carprofen	CPF	NSAIDs	53716-49-7	273.71
Doramectin	DRT	Avermectin	117704-25-3	899.11
Ivermectin	IVT	Avermectin	70288-86-7	875.11
Emamectin	EMT	Avermectin	121124-29-6	886.13
Eprinomectin	EPT	Avermectin	123997-26-2	914.13
Moxidectin	MXT	Avermectin	113507-06-5	639.82
Salinomycin	SNC	Anticoccidial	53003-10-4	751.00
Monensin	MNS	Anticoccidial	17090-79-8	670.87
Narasin	NRS	Anticoccidial	55134-13-9	765.03
Aldrin	ADN	Pesticide	309-00-2	364.91
p,p’-DDT	DDT	Pesticide	50-29-3	354.49
Chlorpyrifos	CPV	Pesticide	2921-88-2	350.59
Dieldrin	DDN	Pesticide	60-57-1	380.91
Dichlorvos	DCV	Pesticide	62-73-7	220.98
Malathion	MLT	Pesticide	121-75-5	330.36
Endosulphan-Sulphate	ESS	Pesticide	1031-07-8	422.92
Chlorfenvinphos	CFP	Pesticide	470-90-6	359.57
Propoxur	PPX	Carbamate	114-26-1	209.24
Carbaryl	CBL	Carbamate	63-25-2	201.23
Propham	PPM	Carbamate	122-42-9	179.22
Methomyl	MTM	Carbamate	16752-77-5	162.21
Pirimicarb	PMC	Carbamate	23103-98-2	238.29
Methiocarb	MTC	Carbamate	2032-65-7	225.31
Ochratoxin A	OCT	Mycotoxin	303-47-9	403.81
Zearalenone	ZRL	Mycotoxin	17924-92-4	318.36

**Table 2:** Established validation parameters for the analytes (limit of detection (LOD), limit of quantification (LOQ), coefficient of determination (*R*^2^) and decision limit (CCα)).

Analyte	Internal Standard	Linear Range (µg kg^−1^)	Coefficient of Determination (*R*^2^)	Spiking Level (µg kg^−1^) at MRL	Mean Recovery (%)	LOD (µg kg^−1^)	LOQ (µg kg^−1^)	CCα (µg kg^−1^)
CTC	TTC-d6	10–200	0.99994	100	92.4	4.71	15.7	115
DXC	TTC-d6	10–200	0.99991	100	78.1	6.57	22.4	116
OTC	TTC-d6	10–200	0.99990	100	83.5	6.34	21.9	116
TTC	TTC-d6	10–200	0.99996	100	94.7	5.96	19.8	113
SFM	SFT-d4	10–200	0.99949	100	99.1	6.35	21.15	117
SFX	SFT-d4	10–200	0.99942	100	103.7	6.10	20.32	117
SFT	SFT-d4	10–200	0.99993	100	98.1	4.36	14.52	118
SFD	SFT-d4	10–200	0.99990	100	100.3	6.39	21.29	112
SFZ	SFT-d4	10–200	0.99986	100	106.8	6.75	22.48	118
SFC	SFP-d4	10–200	0.99991	100	99.6	7.39	24.65	114
SFQ	SFP-d4	10–200	0.99993	100	99.0	5.17	17.24	115
SFP	SFP-d4	10–200	0.99982	100	99.0	5.64	18.80	117
SFN	SFT-d4	10–200	0.99989	100	97.9	5.60	18.66	116
SFL	SFT-d4	10–200	0.99990	100	99.8	4.71	15.71	117
SFO	SFP-d4	10–200	0.99995	100	105.1	8.73	23.18	116
SFA	SFP-4	10–200	0.99993	100	101.0	6.25	19.82	114
LCM	LCM-d3	10–200	0.99995	100	99.1	3.78	12.62	112
ETM	SRM-d3	20–400	0.99997	200	99.0	10.16	33.87	214
TMC	SRM-d3	5–100	0.99989	50	98.0	2.52	8.43	59
TLS	SRM-d3	10–200	0.99992	100	98.6	5.03	12.07	117
SRM	SRM-d3	10–400	0.99995	200	99.4	9.12	30.40	221
GTM	SRM-d3	10–200	0.99993	100	99.1	4.37	14.55	116
TTM	SRM-d3	20–400	0.99990	200	98.8	7.79	25.99	217
CFX	EFX-d5	10–200	0.99988	100	98.9	5.32	12.55	119
DFX	EFX-d5	20–400	0.99991	200	99.5	12.26	29.87	219
DFC	DFC-d5	25–800	0.99997	400	101.0	23.78	38.27	420
EFX	EFX-d5	10–200	0.99991	100	102.0	9.27	14.25	118
FMQ	DFC-d5	20–400	0.99987	200	99.0	13.20	24.16	208
NFX	EFX-d5	10–100	0.99990	50	99.8	9.76	15.86	58
OXA	DFC-d5	25–300	0.99994	150	101.5	14.88	26.37	157
CZL	XLZ-d6	2.5–25	0.99991	12.5	80.0	1.28	4.09	13.8
CPZ	XLZ-d6	2.5–25	0.99994	12.5	87.3	1.30	4.53	14.1
APZ	XLZ-d6	2.5–25	0.99989	12.5	88.2	1.53	4.08	14.2
XLZ	XLZ-d6	2.5–25	0.99997	12.5	88.0	1.42	3.99	13.9
PPZ	XLZ-d6	2.5–25	0.99992	12.5	92.8	1.55	4.46	14.8
AZP	XLZ-d6	10–200	0.99990	100	99.8	6.37	13.97	114
TCZ-SO	TCZ-d3	20–500	0.99994	250	99.8	13.17	23.92	262
CSL	TCZ-d3	5–70	0.99987	35	98.9	3.41	7.03	38
MBZ-NH_2_	MBZ-d3	10–120	0.99983	60	101.8	8.72	11.30	65
OXC	TCZ-d3	2.5–40	0.99991	20	99.3	1.79	4.32	22
FBZ	FBZ-d3	10–100	0.99995	50	99.5	6.54	12.46	58
ABZ	ABZ-d3	10–200	0.99992	100	102.6	11.27	19.97	118
LVS	ABZ-d3	2.5–40	0.99989	20	104.6	1.45	3.64	23
OBZ	ABZ-d3	10–100	0.99986	50	99.4	6.13	11.88	56
TCZ	TCZ-d3	10–200	0.99993	100	99.0	5.05	10.77	114
TBZ	ABZ-d3	10–200	0.99991	100	99.7	5.22	10.42	116
NTX	TCZ-d3	25–800	0.99990	400	99.7	16.33	28.43	418
CST	TCZ-d3	25–1000	0.99993	500	99.6	19.44	32.65	519
RFX	TCZ-d3	5–60	0.99989	30	99.3	5.02	7.80	36
ABZ-SO_2_-NH_2_	ABZ-d3	10–200	0.99994	100	96.8	8.15	15.00	115
TBZ-OH	ABZ-d3	10–200	0.99992	100	99.7	4.98	12.60	113
CPX	CTF-d3	20–400	0.99996	200	101.4	6.89	22.96	215
CFQ	CTF-d3	5–100	0.99987	50	97.9	2.03	6.77	57
CFZ	CTF-d3	5–100	0.99990	50	98.1	2.39	7.99	59
CFN	CTF-d3	5–100	0.99993	50	101.5	2.57	8.56	57
FLX	FLX-d3	2.5–40	0.99995	20	104.6	1.94	4.55	24
MXC	FLX-d3	2.5–40	0.99988	20	99.3	1.30	4.32	23
DCF	FLX-d3	2.5–20	0.99991	10	110.0	1.20	3.96	14
PBZ	FLX-d3	2.5–20	0.99993	10	103.0	1.69	3.85	13
TFA	FLX-d3	5–100	0.99997	50	97.1	2.55	8.51	58
KTF	FLX-d3	2.5–40	0.99989	20	97.5	1.92	4.37	23
IBF	FLX-d3	2.5–40	0.99995	20	95.4	2.04	5.11	23
CPF	FLX-d3	25–1000	0.99990	500	102.1	17.33	28.70	524
DRT	ABZ-d3	5–100	0.99986	50	99.9	5.08	9.88	57
IVT	ABZ-d3	5–40	0.99994	20	99.8	4.56	9.60	24
EMT	ABZ-d3	10–200	0.99990	100	98.1	5.47	18.22	114
EPT	ABZ-d3	10–1000	0.99991	500	101.1	2.76	9.18	523
MXT	ABZ-d3	5–100	0.99988	50	95.8	2.53	8.43	54
SNC	XLZ-d6	2.5–40	0.99996	20	99.1	1.83	3.43	24
MNS	XLZ-d6	2.5–30	0.99991	15	103.9	1.19	3.78	18
NRS	XLZ-d6	2.5–50	0.99989	25	98.2	1.49	4.78	29
ADN	CBL-d7	10–400	0.99992	200	98.2	7.66	14.51	211
DDT	CBL-d7	25–1000	0.99996	500	100.6	12.56	33.50	518
CPV	CBL-d7	10–200	0.99985	100	99.5	4.53	15.08	114
DDN	CBL-d7	10–400	0.99984	200	98.4	5.66	11.89	214
DCV	CBL-d7	2.5–20	0.99991	10	109.0	0.93	2.69	13
MLT	CBL-d7	2.5–40	0.99993	20	99.0	1.74	2.55	23
ESS	CBL-d7	1.5–20	0.99990	10	96.0	0.94	1.98	14
CFP	CBL-d7	1.5–20	0.99995	10	105.3	0.96	3.12	14
PPX	CBL-d7	5–100	0.99989	50	101,7	2.24	7.46	58
CBL	CBL-d7	5–100	0.99992	50	97.7	2.02	6.74	56
PPM	CBL-d7	5–100	0.99991	50	98.0	2.55	7.04	57
MTM	CBL-d7	5–100	0.99996	50	97.9	2.18	7.28	59
PMC	CBL-d7	5–100	0.99988	50	97.1	2.17	7.24	58
MTC	CBL-d7	5–100	0.99990	50	94.4	1.80	5.81	59
OCT	OCT-13C20	2.5–40	0.99993	20	96.96.5	1.93	3.56	23
ZRL	ZRL-13C18	2.5–40	0.99994	20	94.5	2.06	3.98	24

**Table 3:** Precision (repeatability and reproducibility) and accuracy (recovery) values of veterinary drug, pesticide and mycotoxin residues from spiked beef muscle samples.

Analyte	Retention Time (min)	Spiking Level (µg kg^−1^)	Mean Recovery (%)	Repeatability %CV at 1.0 MRL	Acceptable Repeatability Range	Reproducibility %CV at 1.0 MRL	Acceptable Reproducibility Range
CTC	3.47	50	86.20	9.91	≤16.7	10.24	≤25
		100	92.43				
		150	90.52				
DXC	3.54	50	87.33	10.97	≤16.7	11.03	≤25
		100	80.0				
		150	89.08				
OTC	2.89	50	85.87	9.60	≤16.7	9.69	≤25
		100	83.46				
		150	90.12				
TTC	3.07	50	86.90	7.55	≤16.7	7.55	≤25
		100	94.70				
		150	91.94				
SFM	3.45	50	101.70	10.65	≤16.7	11.04	≤25
		100	99.07				
		150	98.95				
SFX	4.29	50	94.78	7.03	≤16.7	8.08	≤25
		100	103.70				
		150	102.00				
SFT	3.68	50	95.89	10.44	≤16.7	12.01	≤25
		100	98.10				
		150	99.64				
SFD	4.55	50	98.34	6.07	≤16.7	7.14	≤25
		100	100.28				
		150	99.10				
SFZ	3.14	50	100.80	8.03	≤16.7	6.95	≤25
		100	106.75				
		150	103.00				
SFC	4.04	50	96.83	8.94	≤16.7	9.00	≤25
		100	99.60				
		150	98.45				
SFQ	4.55	50	101.56	10.06	≤16.7	9.85	≤25
		100	99.02				
		150	98.99				
SFP	3.26	50	94.45	10.80	≤16.7	10.90	≤25
		100	98.96				
		150	97.66				
SFN	3.88	50	93.06	11.45	≤16.7	11.30	≤25
		100	97.90				
		150	98.20				
SFL	3.59	50	97.00	9.89	≤16.7	10.40	≤25
		100	99.76				
		150	99.50				
SFO	3.89	50	94.66	6.05	≤16.7	6.93	≤25
		100	105.10				
		150	101.00				
SFA	3.11	50	98.00	8.86	≤16.7	8.02	≤25
		100	101.00				
		150	99.97				
LCM	1.54	50	96.57	8.00	≤16.7	8.10	≤25
		100	99.05				
		150	99.00				
ETM	3.94	100	99.60	11.23	≤14.7	10.93	≤22
		200	98.00				
		300	105.78				
TMC	3.50	25	94.00	5.45	≤16.7	6.13	≤25
		50	97.96				
		75	98.62				
TLS	3.98	50	95.00	11.23	≤16.7	11.15	≤25
		100	98.61				
		150	99.94				
SRM	3.22	100	100.80	10.54	≤14.7	13.35	≤22
		200	99.35				
		300	99.60				
GTM	3.36	50	100.98	9.33	≤16.7	10.22	≤25
		100	99.05				
		150	98.55				
TTM	2.78	100	99.22	8.67	≤14.7	12.07	≤22
		200	98.75				
		300	103.00				
CFX	2.93	50	98.33	12.91	≤16.7	12.01	≤25
		100	98.90				
		150	104.10				
DFX	3.02	100	95.00	11.09	≤14.7	12.05	≤22
		200	99.50				
		300	96.70				
DFC	3.31	200	89.00	7.99	≤14.7	9.68	≤22
		400	101.00				
		600	106.60				
EFX	3.07	50	94.35	9.78	≤16.7	9.80	≤25
		100	102.00				
		150	98.17				
FMQ	5.84	100	105.20	7.77	≤14.7	8.09	≤22
		200	98.90				
		300	97.00				
NFX	2.83	25	88.00	4.67	≤16.7	5.03	≤25
		50	99.78				
		75	96.50				
OXA	4.42	75	92.30	9.66	≤14.7	10.08	≤22
		150	101.53				
		225	99.30				
CZL	3.55	6.25	84.33	3.02	≤16.7	2.30	≤25
		12.5	80.00				
		18.75	94.00				
CPZ	4.30	6.25	85.40	1.89	≤16.7	1.98	≤25
		12.5	87.20				
		18.75	93.00				
APZ	4.01	6.25	90.30	1.78	≤16.7	1.92	≤25
		12.5	88.20				
		18.75	96.00				
XLZ	3.17	6.25	92.50	1.75	≤16.7	1.74	≤25
		12.5	88.00				
		18.75	98.55				
PPZ	5.42	6.25	88.23	1.88	≤16.7	1.95	≤25
		12.5	92.80				
		18.75	95.40				
AZP	2.63	50	89.78	9.83	≤16.7	9.04	≤25
		100	99.80				
		150	102.45				
TCZ-SO	5.45	125	90.00	7.93	≤14.7	8.18	≤22
		250	99.84				
		500	100.90				
CSL	4.37	17.50	89.50	2.44	≤16.7	2.05	≤25
		35	98.86				
		52.50	97.40				
MBZ-NH_2_	3.32	30	94.00	2.76	≤16.7	2.95	≤25
		60	101.72				
		90	98.30				
OXC	5.64	10	102.80	1.56	≤16.7	1.44	≤25
		20	99.30				
		30	98.56				
FBZ	4.95	25	95.74	4.79	≤16.7	5.06	≤25
		50	99.50				
		75	96.00				
ABZ	4.47	50	84.32	9.87	≤16.7	10.0	≤25
		100	102.00				
		150	98.44				
LVS	2.30	10	95.50	1.50	≤16.7	1.69	≤25
		20	102.00				
		30	99.10				
OBZ	3.96	25	94.90	4.13	≤16.7	4.12	≤25
		50	99.40				
		75	97.72				
TCZ	5.67	50	98.60	9.00	≤16.7	9.35	≤25
		100	98.96				
		150	106.00				
TBZ	2.56	50	98.08	11.06	≤16.7	10.01	≤25
		100	99.70				
		150	102.20				
NTX	5.47	200	89.80	11.15	≤14.7	11.40	≤22
		400	99.73				
		600	99.50				
CST	8.00	250	98.00	12.55	≤14.7	12.50	≤22
		500	99.68				
		750	95.60				
RFX	9.84	15	94.08	2.33	≤16.7	2.30	≤25
		30	99.33				
		45	103.20				
ABZ-SO_2_-NH_2_	1.56	50	98.00	10.09	≤16.7	10.95	≤25
		100	96.80				
		150	99.22				
TBZ-OH	1.55	50	89.70	8.17	≤16.7	8.00	≤25
		100	99.70				
		150	98.46				
CPX	2.66	100	95.60	7.65	≤14.7	7.69	≤22
		200	101.40				
		300	99.00				
CFQ	4.20	25	88.54	5.11	≤16.7	5.02	≤25
		50	97.94				
		75	99.40				
CFZ	3.68	25	89.90	6.25	≤16.7	6.27	≤25
		50	98.10				
		75	99.50				
CFN	2.93	25	98.00	4.25	≤16.7	4.08	≤25
		50	101.50				
		75	102.00				
FLX	5.39	10	93.20	1.77	≤16.7	1.74	≤25
		20	104.65				
		30	100.86				
MXC	5.27	10	94.10	2.32	≤16.7	2.09	≤25
		20	99.90				
		30	102.00				
DCF	5.45	5	104.06	1.60	≤16.7	1.67	≤25
		10	109.80				
		15	106.00				
PBZ	5.57	5	95.12	1.90	≤16.7	1.83	≤25
		10	102.90				
		15	98.28				
TFA	5.81	25	89.96	6.57	≤16.7	6.00	≤25
		50	97.10				
		75	95.42				
KTF	5.15	10	95.30	3.00	≤16.7	2.38	≤25
		20	97.50				
		30	96.00				
IBF	5.78	10	93.00	2.31	≤16.7	2.15	≤25
		20	95.40				
		30	97.00				
CPF	5.29	250	94.33	7.98	≤14.7	8.07	≤22
		500	102.12				
		750	97.00				
DRT	2.93	25	94.00	4.88	≤16.7	5.09	≤25
		50	97.92				
		75	100.54				
IVT	2.47	10	90.04	2.67	≤16.7	2.60	≤25
		20	99.90				
		30	107.40				
EMT	6.16	50	95.02	10.23	≤16.7	9.75	≤25
		100	98.10				
		150	97.00				
EPT	7.30	250	98.00	10.75	≤14.7	10.72	≤22
		500	101.12				
		750	100.96				
MXT	9.33	25	87.04	3.44	≤16.7	3.83	≤25
		50	95.80				
		75	96.43				
SNC	7.37	10	90.35	2.65	≤16.7	2.65	≤25
		20	99.10				
		30	94.52				
MNS	7.78	7.50	93.07	1.67	≤16.7	1.49	≤25
		15	103.87				
		22.50	98.00				
NRS	9.23	12.50	96.40	3.55	≤16.7	3.06	≤25
		25	98.24				
		37.50	97.31				
ADN	1.54	100	101.75	8.66	≤14.7	8.68	≤22
		200	98.20				
		300	99.69				
DDT	6.74	250	91.08	8.90	≤14.7	8.96	≤22
		500	100.60				
		750	94.26				
CPV	5.73	50	90.00	7.96	≤16.7	9.13	≤25
		100	99.50				
		150	93.52				
DDN	1.94	100	87.90	10.53	≤14.7	10.45	≤22
		200	98.34				
		300	92.47				
DCV	4.89	5	94.85	1.55	≤16.7	1.55	≤25
		10	108.80				
		15	105.00				
MLT	5.51	10	88.48	2.00	≤16.7	2.05	≤25
		20	99.00				
		30	95.63				
ESS	5.53	5	93.44	2.47	≤16.7	2.60	≤25
		10	96.00				
		15	90.94				
CFP	5.72	5	96.78	1.90	≤16.7	1.97	≤25
		10	105.30				
		15	97.04				
PPX	5.00	25	91.89	4.12	≤16.7	4.08	≤25
		50	101.68				
		75	98.00				
CBL	5.04	25	95.77	4.00	≤16.7	4.10	≤25
		50	97.72				
		75	98.54				
PPM	5.18	25	92.30	4.77	≤16.7	4.76	≤25
		50	98.00				
		75	95.95				
MTM	5.33	25	89.06	5.52	≤16.7	5.90	≤25
		50	97.94				
		75	99.04				
PMC	3.51	25	96.00	6.69	≤16.7	6.14	≤25
		50	97.10				
		75	100.60				
MTC	5.35	25	93.72	7.85	≤16.7	7.30	≤25
		50	94.36				
		75	90.81				
OCT	5.27	10	90.56	2.55	≤16.7	2.35	≤25
		20	96.50				
		30	95.84				
ZRL	5.33	10	92.04	2.76	≤16.7	2.84	≤25
		20	94.50				
		30	98.59				

**Table 4:** Greenness profile of the proposed method using five different green analytical chemistry (GAC) metrics.

Applied Instrument and Chromatographic Conditions	Analytical Eco-Scale (AES) Assessment (Penalty Points)	NEMI	GAPI	AGREE	ComplexGAPI
**Analytical method**: LC–MS/MS method for the analysis of contaminants in beef muscle **Mobile phase**: (A): 0.1% formic acid in H_2_O (B): 0.1% formic acid in acetonitrile **Flow rate**: 0.550 mL min^−1^ **Analysis time**: 13 min	**Reagents** Water (0) Formic acid (6) Acetonitrile (4) **Instruments** LC–MS/MS (2) **Occupational hazards** Waste (3) **Total penalty points**: 15 **AES score**: 85 **Remarks**: Excellent green method	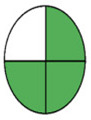	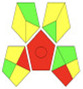	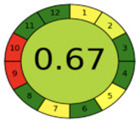	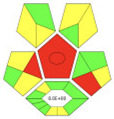

## Data Availability

The original contributions presented in this study are included in the article/Supplementary Materials. Further inquiries can be directed to the corresponding author.

## Supplementary Materials

The following supporting information can be downloaded at: https://www.mdpi.com/article/10.3390/foods14050720/s1, Figure S1: Effect of ionic liquid solvent volume on extraction recovery (blank extracts: 5.0 mL; spiking level: 1 MRL; pH 6; disperser solvent (acetonitrile): 0.5 mL; extraction time: 1.0 min; centrifugation time: 5.0 min; ionic liquid ([C_8_MIm[PF_6_]): 40, 50, 60, 70, 80, 90 µL. Figure S2: Effect of disperser solvent volume on extraction recovery (blank extracts: 5.0 mL; spiking level: 1 MRL; pH 6; ionic liquid ([C_8_MIm][PF_6_]): 60 µL; extraction time: 1.0 min; centrifugation time: 5.0 min; disperser solvent (acetonitrile): 0.3, 0.4, 0.5, 0.6, 0.8, 1.0 mL. Figure S3: Effect of salt (NaCl) addition on extraction recovery (blank extracts: 5.0 mL; spiking level: 1 MRL; disperser solvent (acetonitrile): 0.4 mL; ionic liquid ([C_8_MIm][PF_6_]): 60 µL; pH 6; extraction time: 1.0 min; centrifugation time: 5.0 min; %NaCl (w/v): 0, 0.5, 1, 2, 4, 6). Figure S4: Effect of extraction time on extraction recovery (blank extracts: 5.0 mL; pH 6; spiking level: 1 MRL; disperser solvent (acetonitrile): 0.4 mL; ionic liquid ([C_8_MIm][PF_6_]): 60 µL; centrifugation time: 5.0 min; extraction time: 3.0, 5.0, 10.0, 20.0, 30.0, 40.0, 50.0, 60.0 s). Table S1: LC elution programme. Table S2: Ion source and gas parameters. Table S3: Comparison of IL–DLLME method with QuEChERS methods. Table S4: Comparison of IL–DLLME and QuEChERS methods in terms of time, resources and costs (Tshepho et al., 2023) [25]. Table S5: Description and colour coding of GAPI parameters for greenness assessment of IL–DLLME/LC–MS/MS method. Table S6: Description and colour coding of ComplexGAPI parameters for greenness assessment of IL–DLLME/LC–MS/MS method.
