# Supplementary material for: Multiclass Determination of 87 Mixed Veterinary Drugs, Pesticides and Mycotoxin Residues in Beef Muscle Samples by Ionic Liquid-Based Dispersive Liquid–Liquid Microextraction and Liquid Chromatography Tandem Mass Spectrometry
Source: Foods. 2025 Feb 20;14(5):720. doi: 10.3390/foods14050720 (PMC11898575; doi:10.3390/foods14050720)
Supplement: Supplementary file 1 [file foods-14-00720-s001.zip › foods-3371477-supplementary.pdf]

Supplementary Materials:

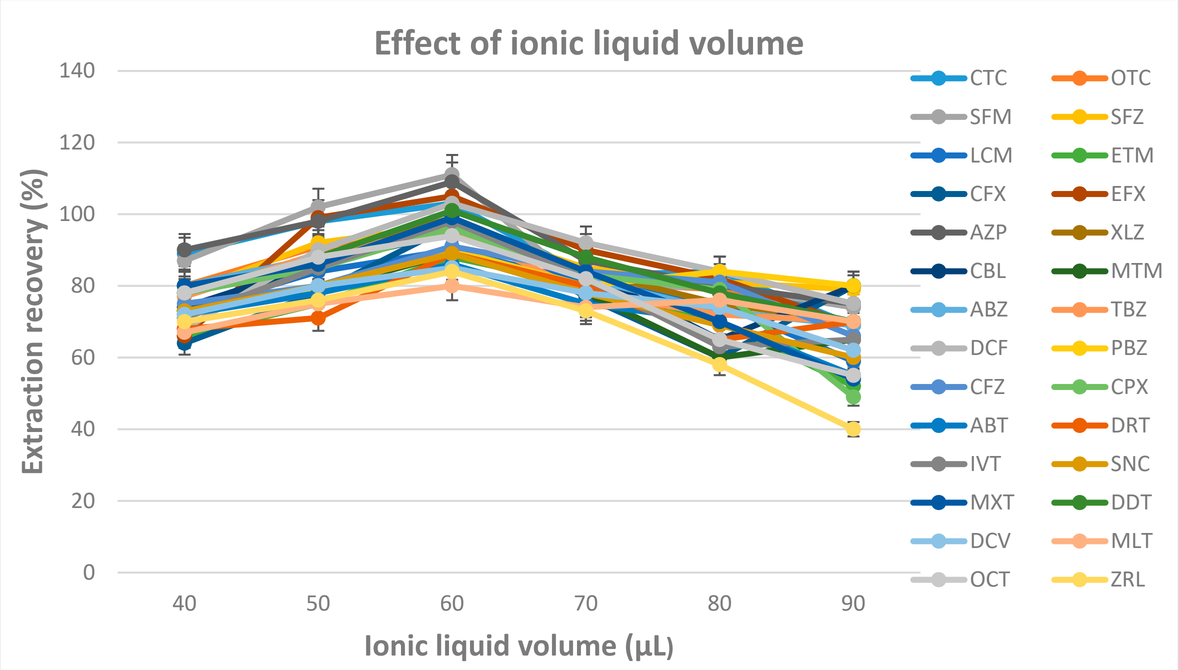

Figure S1: *Effect of ionic liquid solvent volume on extraction recovery (blank extracts: 5.0 mL; spiking level: 1 MRL; pH 6; disperser solvent (acetonitrile): 0.5 mL; extraction time: 1.0 min; centrifugation time: 5.0 min; ionic liquid ([C_8MIm[PF_6]): 40, 50, 60, 70, 80, 90 μL)*

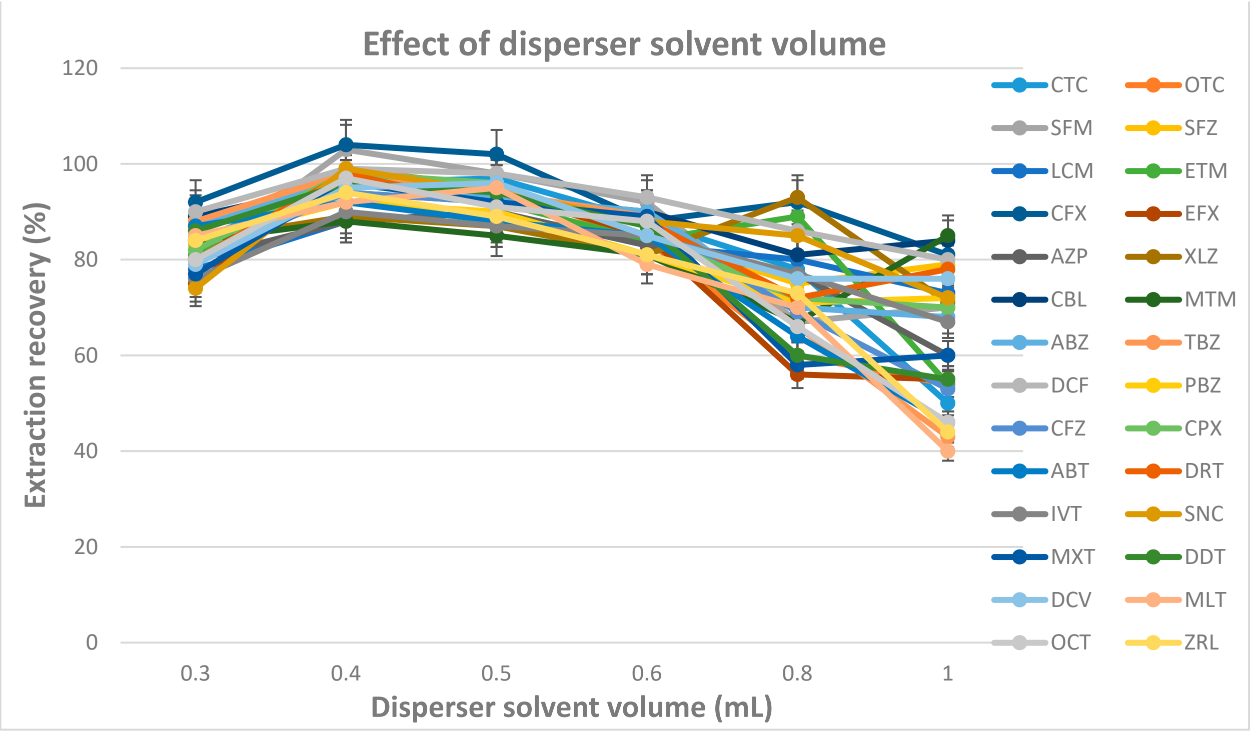

Figure S2: Effect of disperser solvent volume on extraction recovery (blank extracts: 5.0 mL; spiking level: 1 MRL; pH 6; ionic liquid ([C_8MIm][PF_6]): 60 µL; extraction time: 1.0 min; centrifugation time: 5.0 min; disperser solvent (acetonitrile): 0.3, 0.4, 0.5, 0.6, 0.8, 1.0 mL)

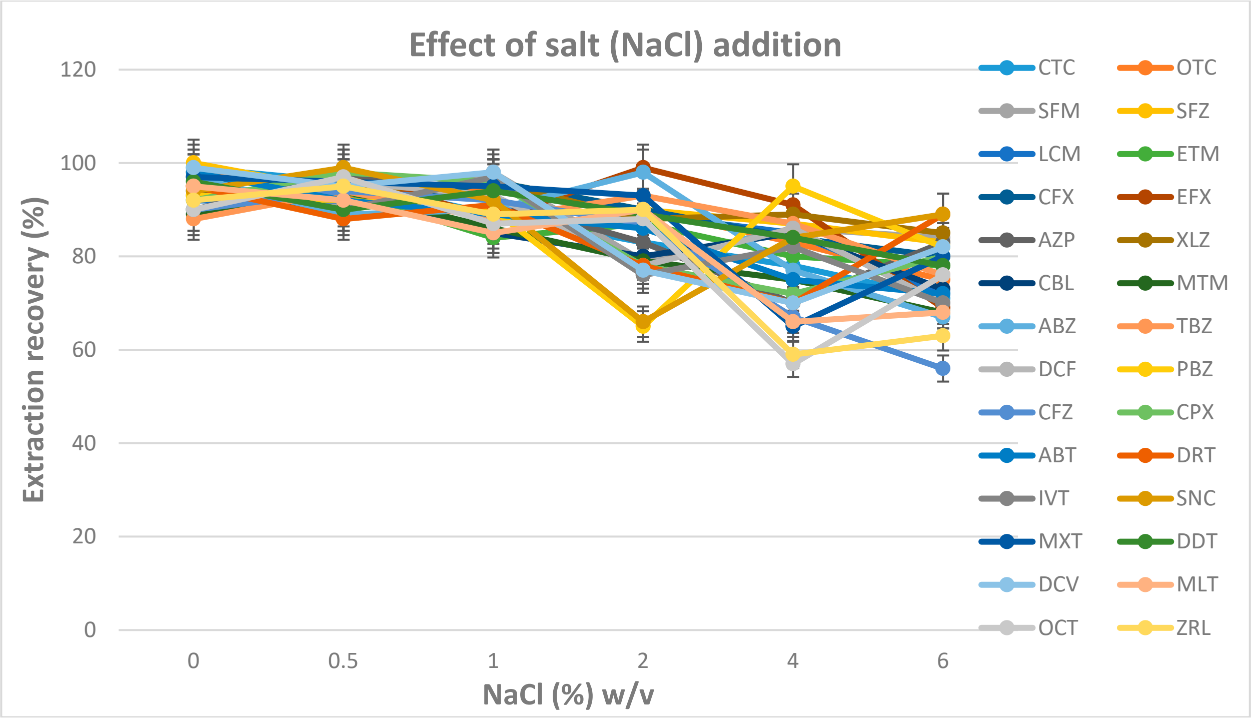

Figure S3: *Effect of salt (NaCl) addition on extraction recovery (blank extracts: 5.0 mL; spiking level: 1 MRL; disperser solvent (acetonitrile): 0.4 mL; ionic liquid ([C_8MIm][PF_6]): 60 µL; pH 6; extraction time: 1.0 min; centrifugation time: 5.0 min; %NaCl (w/v): 0, 0.5, 1, 2, 4, 6)*

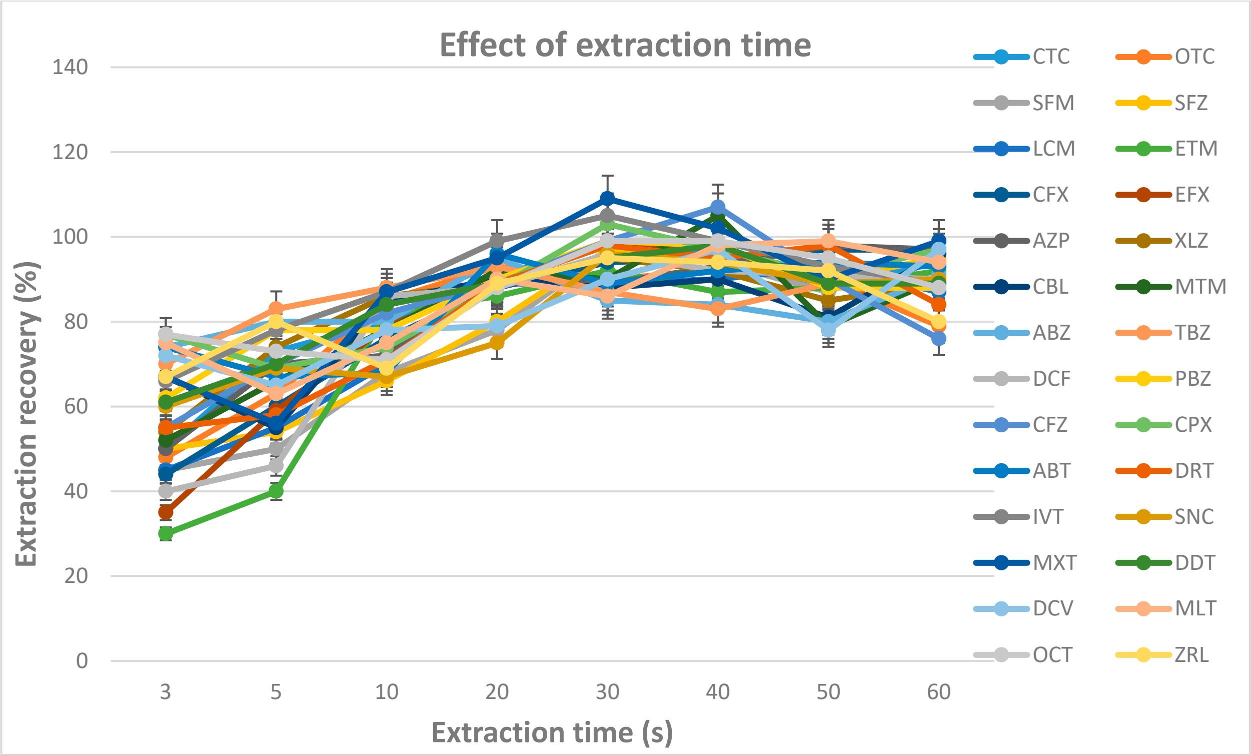

Figure S4: Effect of extraction time on extraction recovery (blank extracts: 5.0 mL; pH 6; spiking level: 1 MRL; disperser solvent (acetonitrile): 0.4 mL; ionic liquid ([C_8MIm][PF_6]): 60 µL; centrifugation time: 5.0 min; extraction time: 3.0, 5.0, 10.0, 20.0, 30.0, 40.0, 50.0, 60.0 s)

Table S1: LC elution programme

Time (min)	Flow rate (mL min^{-1})	A (%)	B (%)
0.00	0.550	90	10
1.00	0.550	90	10
3.00	0.550	70	30
8.00	0.550	90	10
13.00	0.550	90	10

Table S2: Ion source and gas parameters

Ion source and gas parameters	Optimum value (positive/negative mode)
Ion source voltage (IV)	5 000/-4 000
Temperature (Temp)	500
Ion source gas 1 (GS1)	50/40
Ion source gas 2 (GS2)	60/50
Curtain gas (CUR)	40/40
Collision gas (CAD)	Medium/Medium
Entrance potential (EP)	10/-10

Table S3: Comparison of IL–DLLME method with QuEChERS methods

Analyte	Method	Mean recovery (%)			Standard deviation	t-value	t-critical value
		0.5 x MRL	1.0 x MRL	1.5 x MRL			
CTC	IL-DLLME	86.20	92.43	90.52	4.84	-1.23	4.30
	QuEChERS	96.00	94.00	97.00			
DXC	IL-DLLME	87.33	78.14	89.08	8.23	-1.36	4.30
	QuEChERS	96.00	94.00	98.00			
OTC	IL-DLLME	85.87	83.46	90.12	8.04	-1.06	4.30
	QuEChERS	99.00	98.00	88.00			
TTC	IL-DLLME	86.90	94.70	91.94	4.80	-1.35	4.30
	QuEChERS	96.00	99.00	98.00			
SFM	IL-DLLME	101.70	99.07	98.95	1.03	-0.75	4.30
	QuEChERS	104.00	98.33	99.70			
SFX	IL-DLLME	94.78	103.70	102.00	2.37	0.96	4.30
	QuEChERS	96.00	99.66	98.00			
SFT	IL-DLLME	95.89	98.10	99.64	1.56	0.43	4.30
	QuEChERS	98.13	95.46	98.00			
SFD	IL-DLLME	98.34	100.28	99.10	1.50	1.37	4.30
	QuEChERS	97.00	98.00	96.55			
SFZ	IL-DLLME	100.80	106.75	103.00	2.08	1.39	4.30
	QuEChERS	97.45	104.52	99.87			
SFC	IL-DLLME	96.83	99.60	98.45	2.74	1.16	4.30
	QuEChERS	95.00	93.30	97.00			
SFQ	IL-DLLME	101.56	99.02	98.99	1.49	1.34	4.30
	QuEChERS	99.10	98.00	96.50			
SFP	IL-DLLME	94.45	98.96	97.66	2.26	0.85	4.30
	QuEChERS	90.04	96.00	99.24			
SFN	IL-DLLME	93.06	97.90	98.20	1.05	0.67	4.30
	QuEChERS	91.08	99.00	96.98			
SFL	IL-DLLME	97.00	99.76	99.50	1.47	1.36	4.30
	QuEChERS	95.55	98.00	96.70			
SFO	IL-DLLME	94.66	105.10	101.00	1.43	0.70	4.30
	QuEChERS	96.00	102.20	99.54			
SFA	IL-DLLME	98.00	101.00	99.97	0.80	1.26	4.30
	QuEChERS	97.10	100.55	98.30			
LCM	IL-DLLME	96.57	99.05	99.00	1.15	-0.20	4.30
	QuEChERS	95.00	101.40	98.90			
ETM	IL-DLLME	99.60	98.00	105.78	2.09	0.57	4.30
	QuEChERS	96.84	100.60	102.33			
TMC	IL-DLLME	94.00	97.96	98.62	1.31	-1.16	4.30
	QuEChERS	96.44	98.00	100.70			
TLS	IL-DLLME	95.00	98.61	99.94	1.15	0.45	4.30
	QuEChERS	96.67	97.00	98.34			
SRM	IL-DLLME	100.80	99.35	99.60	2.74	1.38	4.30
	QuEChERS	98.10	95.30	94.99			
GTM	IL-DLLME	100.98	99.05	98.55	3.49	0.41	4.30
	QuEChERS	95.40	104.22	94.67			

Analyte	Method	Mean recovery (%)			Standard deviation	t-value	t-critical value
		0.5 x MRL	1.0 x MRL	1.5 x MRL			
TTM	IL-DLLME	99.22	98.75	103.00	2.28	1.32	4.30
	QuEChERS	97.88	95.08	99.00			
CFX	IL-DLLME	98.33	98.90	104.10	2.46	1.26	4.30
	QuEChERS	96.25	97.04	98.77			
DFX	IL-DLLME	95.00	99.50	96.70	2.02	-0.60	4.30
	QuEChERS	93.00	103.44	98.93			
DFC	IL-DLLME	89.00	101.00	106.60	3.80	1.33	4.30
	QuEChERS	84.50	97.84	99.10			
EFX	IL-DLLME	94.35	102.00	98.17	2.05	0.12	4.30
	QuEChERS	98.09	99.00	96.67			
FMQ	IL-DLLME	105.20	98.90	97.00	1.67	0.91	4.30
	QuEChERS	102.00	96.55	98.00			
NFX	IL-DLLME	88.00	99.78	96.50	1.60	-0.64	4.30
	QuEChERS	90.86	98.00	98.52			
OXA	IL-DLLME	92.30	101.53	99.30	1.42	0.45	4.30
	QuEChERS	94.34	99.81	97.05			
CZL	IL-DLLME	84.33	80.00	94.00	3.88	-1.16	4.30
	QuEChERS	85.3	88.56	98.04			
APZ	IL-DLLME	90.30	88.20	96.00	3.11	-0.71	4.30
	QuEChERS	92.66	94.97	93.45			
XLZ	IL-DLLME	92.50	88.00	98.55	2.18	-1.37	4.30
	QuEChERS	95.00	92.10	100.86			
PPZ	IL-DLLME	88.23	92.80	95.40	2.47	-0.24	4.30
	QuEChERS	84.00	96.40	97.82			
AZP	IL-DLLME	89.78	99.80	102.45	3.46	1.40	4.30
	QuEChERS	83.90	95.57	98.04			
TCZ-SO	IL-DLLME	90.00	99.84	100.90	1.81	1.34	4.30
	QuEChERS	86.44	98.03	99.00			
CSL	IL-DLLME	89.50	98.86	97.40	2.29	-0.31	4.30
	QuEChERS	88.30	104.02	95.58			
MBZ-NH2	IL-DLLME	94.00	101.72	98.30	1.97	-0.47	4.30
	QuEChERS	96.65	98.93	101.20			
OXC	IL-DLLME	102.80	99.30	98.56	3.46	1.33	4.30
	QuEChERS	98.77	92.41	95.62			
FBZ	IL-DLLME	95.74	99.50	96.00	2.71	1.38	4.30
	QuEChERS	90.96	96.74	92.33			
ABZ	IL-DLLME	84.32	102.00	98.44	2.06	-1.02	4.30
	QuEChERS	88.70	104.43	97.90			
LVS	IL-DLLME	95.50	102.00	99.10	2.20	-0.68	4.30
	QuEChERS	97.67	99.85	103.54			
OBZ	IL-DLLME	94.90	99.40	97.72	1.33	0.02	4.30
	QuEChERS	96.34	100.58	95.04			
TCZ	IL-DLLME	98.60	98.96	106.00	4.33	0.31	4.30
	QuEChERS	105.00	96.75	97.83			
TBZ	IL-DLLME	98.08	99.70	102.20	3.28	1.40	4.30
	QuEChERS	93.27	94.40	98.56			

Analyte	Method	Mean recovery (%)			Standard deviation	t-value	t-critical value
		0.5 x MRL	1.0 x MRL	1.5 x MRL			
NTX	IL-DLLME	89.80	99.73	99.50	3.01	1.41	4.30
	QuEChERS	86.00	95.49	94.80			
CST	IL-DLLME	98.00	99.68	95.60	2.85	-0.03	4.30
	QuEChERS	100.51	94.08	98.94			
RFX	IL-DLLME	94.08	99.33	103.20	2.74	-0.07	4.30
		97.90	101.23	98.02			
ABZ-SO-NH2	IL-DLLME	98.00	96.80	99.22	2.22	-0.76	4.30
	QuEChERS	103.00	95.34	100.76			
TBZ-OH	IL-DLLME	89.70	99.70	98.46	3.08	0.10	4.30
	QuEChERS	94.00	93.56	99.38			
CPX	IL-DLLME	95.60	101.4	99.00	2.51	0.46	4.30
	QuEChERS	92.00	97.95	102.60			
CFQ	IL-DLLME	88.54	97.94	99.40	1.75	-0.09	4.30
	QuEChERS	90.70	99.57	96.08			
CFZ	IL-DLLME	89.90	98.10	99.50	3.48	0.74	4.30
	QuEChERS	84.57	92.36	102.88			
CFN	IL-DLLME	98.00	101.50	102.00	3.26	-0.65	4.30
	QuEChERS	94.52	107.66	105.71			
FLX	IL-DLLME	93.20	104.65	100.86	2.02	0.10	4.30
	QuEChERS	97.00	102.90	98.20			
MXC	IL-DLLME	94.10	99.90	102.00	1.99	0.51	4.30
	QuEChERS	96.74	97.66	98.58			
DCF	IL-DLLME	104.06	109.80	106.00	7.97	1.41	4.30
	QuEChERS	94.00	98.48	93.68			
PBZ	IL-DLLME	95.12	102.90	98.28	1.83	0.55	4.30
	QuEChERS	97.44	99.82	96.00			
TFA	IL-DLLME	89.96	97.10	95.42	2.06	0.60	4.30
	QuEChERS	86.78	99.62	92.35			
KTF	IL-DLLME	95.30	97.50	96.00	2.79	0.41	4.30
	QuEChERS	89.80	95.88	99.70			
IBF	IL-DLLME	93.00	95.40	97.00	2.43	0.50	4.30
	QuEChERS	91.00	98.45	92.30			
CPF	IL-DLLME	94.33	102.12	97.00	2.32	0.48	4.30
	QuEChERS	95.33	96.64	98.14			
DRT	IL-DLLME	94.00	97.92	100.54	2.08	-0.14	4.30
	QuEChERS	97.00	99.56	96.77			
IVT	IL-DLLME	90.04	99.90	107.4	3.56	-0.47	4.30
	QuEChERS	94.00	105.89	102.44			
EMT	IL-DLLME	95.02	98.10	97.00	3.77	0.80	4.30
	QuEChERS	86.00	96.73	98.40			
EPT	IL-DLLME	98.00	101.12	100.96	2.53	1.24	4.30
	QuEChERS	96.45	98.83	95.42			
MXT	IL-DLLME	87.04	95.80	96.43	2.28	-0.83	4.30
	QuEChERS	92.00	94.37	98.56			
SNC	IL-DLLME	90.35	99.10	94.52	2.54	-1.32	4.30

Analyte	Method	Mean recovery (%)			Standard deviation	t-value	t-critical value
		0.5 x MRL	1.0 x MRL	1.5 x MRL			
	QuEChERS	93.12	104.23	96.70			
MNS	IL-DLLME	93.07	103.87	98.00	2.31	1.29	4.30
	QuEChERS	90.77	99.00	96.24			
NRS	IL-DLLME	96.40	98.24	97.31	1.42	0.22	4.30
	QuEChERS	94.00	97.36	99.66			
ADN	IL-DLLME	101.75	98.20	99.69	1.30	0.92	4.30
	QuEChERS	99.55	96.04	100.48			
DDT	IL-DLLME	91.08	100.60	94.26	2.50	0.52	4.30
	QuEChERS	88.30	96.22	97.50			
CPV	IL-DLLME	90.00	99.50	93.52	1.95	0.65	4.30
	QuEChERS	91.49	98.70	89.04			
DDN	IL-DLLME	87.90	98.34	92.47	1.93	0.45	4.30
	QuEChERS	90.10	94.22	91.80			
DCV	IL-DLLME	94.85	108.80	105.00	3.40	0.99	4.30
	QuEChERS	96.33	102.78	99.45			
MLT	IL-DLLME	88.48	99.00	95.63	2.05	0.14	4.30
	QuEChERS	84.12	100.55	97.60			
ESS	IL-DLLME	93.44	96.00	90.94	3.67	1.18	4.30
	QuEChERS	85.44	92.00	89.98			
CFP	IL-DLLME	96.78	105.30	97.04	2.42	0.77	4.30
	QuEChERS	98.31	99.80	95.44			
PPX	IL-DLLME	91.89	101.68	98.00	1.68	0.39	4.30
	QuEChERS	94.22	98.46	96.90			
CBL	IL-DLLME	95.77	97.72	98.54	1.24	-0.10	4.30
	QuEChERS	94.50	100.30	97.60			
PPM	IL-DLLME	92.30	98.00	95.95	2.28	0.57	4.30
	QuEChERS	86.90	99.42	96.00			
MTM	IL-DLLME	89.06	97.94	99.04	1.56	-0.10	4.30
	QuEChERS	88.70	101.00	96.79			
PMC	IL-DLLME	96.00	97.10	100.60	5.58	1.00	4.30
	QuEChERS	90.00	98.60	88.40			
MTC	IL-DLLME	93.72	94.36	90.81	1.35	-0.51	4.30
	QuEChERS	95.40	92.55	93.00			
OCT	IL-DLLME	90.56	96.50	95.84	-	-	-
	-	-	-	-			
ZRL	IL-DLLME	92.04	94.50	98.59	-	-	-
	-	-	-	-			

Table S4: Comparison of IL–DLLME and QuEChERS methods in terms of time, resources and costs (Tshepho et al., 2023) [25]

Time, resources and cost	IL–DLLME	QuEChERS
Time for weighing (20 samples)	1 h	1 h
Time for weighing salts	-	2 h
Sample preparation time	1 day	1.5 days
Centrifugation time (pre-treatment + extraction)	10 min+5 min = 15 min	10 min+5 min = 15 min
Volume of acetonitrile (pre-treatment + extraction)	6 mL + 0.4 mL	8 mL + 0.4 mL
Volume of water (pretreatment)	2 mL	2 mL
Drying time (20 samples)	1 h	1.5 h
NaCl per sample	-	1 g
$MgSO_4$ per sample	-	5 g solvent extraction + 1 g dispersive SPE
C18 sorbent per sample	-	500 mg
$[C_8MIm][PF_6]$ per sample	0.06 mL	-
DMSO	0.2 mL	0.2 mL
Reagents going to waste (20 samples)	Acetonitrile (60 mL)	• Acetonitrile (100 mL)<br>• $MgSO_4$ (120 g)<br>• C18 sorbent (10 g)
Estimated costs of reagents/year at Botswana National Veterinary Laboratory	1 x 50 g $[C_8MIm][PF_6]$ (1 044 USD)<br>Enough to be used for 2 years	• 2 x 200 g sorbent (800 USD)<br>• 3 x 500 g $MgSO_4$ (500 USD)<br>• 1 x 250 g NaCl (70 USD)<br>Estimated total costs = 1 370 USD

Table S5: Description and colour coding of GAPI parameters for greenness assessment of IL–DLLME/LC–MS/MS method

Category	Description	Colour coding
**Sample preparation**		
Collection (1)	Off-line	Red
Preservation (2)	Chemical or physical	Yellow
Transport (3)	None	Green
Storage (4)	Normal conditions	Yellow
Type of method (5)	Extraction required	Red
Scale of extraction (6)	Microextraction (IL-DLLME)	Yellow
Solvents/reagents used (7)	Green solvents/reagents used	Yellow
Additional treatments (8)	Simple treatment	Yellow
**Reagents and solvents**		
Amount (9)	< 10 mL	Green
Health hazard (10)	Acetonitrile (Moderately toxic; NFPA = 2)<br>Formic acid (Moderately toxic; NFPA = 3)	Yellow<br>Yellow
Safety hazard (11)	Acetonitrile (Highest NFPA score of 3 flammability)<br>Formic acid (Highest NFPA flammability score of 2)	Yellow<br>Yellow
**Instrumentation**		
Energy (12)	⩽ 1.5 kWh per sample	Yellow
Occupational hazard (13)	Hermitization of analytical process	Green
Waste (14)	1-10 mL	yellow
Waste treatments (15)	No treatment	Red

Table S6: Description and colour coding of ComplexGAPI parameters for greenness assessment of IL–DLLME/LC–MS/MS method

	Category	Description	Colour coding
	**Sample preparation**		
1	Collection	Off-line	Red
2	Preservation	Chemical or physical	Yellow
3	Transport	Not required	Green
4	Storage	Normal conditions	Yellow
5	Type of method	Extraction required	Red
6	Scale of extraction	Microextraction (IL-DLLME)	Yellow
7	Solvents/reagents used	Green solvents/reagents used	Green
8	Additional treatments	None	Green
	**Reagents and solvents**		
9	Amount	< 10 mL	Green
10	Health hazard	Acetonitrile (Moderately toxic; NFPA = 2)	Yellow
11	Safety hazard	Acetonitrile (Highest NFPA score of 3 flammability)	Yellow
	**Instrumentation**		
12	Energy	⩽ 1.5 kWh per sample	Yellow
13	Occupational hazard	Hermitization of analytical process	Green
14	Waste	1-10 mL	Yellow
15	Waste treatments	Degradation, passivation	Yellow
	**Category**	**Description**	**Colour coding**
	**Methods**		
16	Type of analysis	Qualitative and quantitative	Red
	**Category**	**Description**	**Colour coding**
	**PRE-ANALYSIS PROCESSES**		
	**Yield and conditions**		
I	Yield	70 – 89%	Yellow
II	Temperature	Room tempearature, < 1 h	Green
	**Relationship to Green Economy**		
III	Number of rules met	3 - 4	Yellow
	**Reagents and solvents**		
IVa	Health hazard	Moderately toxic, NFPA = 2 or 3	Yellow
IVb	Safety hazard	Highest NFPA flammability or instability score is 2 or 3	Yellow
	**Intrumentation**		
Va	Technical setup	Common setup	Green
Vb	Energy	≤ 0.1 kWk per sample	Green
Vc	Occupational hazard	Hermatization of analytical process	Green
	**Workup and purification**		
VIa	End products Workup, purification	None or simple process	Grren
VIb	Purity	97 - 98	Yellow
	**E-factor**		
VII	E-factor impact		
